# Development of a generalized pseudo-probabilistic approach for characterizing ecological conditions in estuaries using secondary data

**DOI:** 10.1007/s10661-024-12877-8

**Published:** 2024-07-20

**Authors:** Linda C. Harwell, Courtney A. McMillion, Andrea M. Lamper, J. Kevin Summers

**Affiliations:** 1 US Environmental Protection Agency, 1 Sabine Island Drive, Gulf Breeze, FL 32561 USA; 2 Oak Ridge Associated Universities, 1 Sabine Island Drive, Gulf Breeze, FL 32561 USA; 3 Santa Rosa County Florida, Development Services Department, 6051 Old Bagdad Highway, Suite 202, Milton, FL 32583 USA; 4 CDM Smith, 670 N Commercial St, Unit 208, Manchester, NH 03101 USA

**Keywords:** Assessment, Estuary, Probabilistic, Secondary, NCCA

## Abstract

Under the best circumstances, achieving or sustaining optimum ecological conditions in estuaries is challenging. Persistent information gaps in estuarine data make it difficult to differentiate natural variability from potential regime shifts. Long-term monitoring is critical for tracking ecological change over time. In the United States (US), many resource management programs are working at maximum capacity to address existing state and federal water quality mandates (e.g., pollutant load limits, climate impact mitigation, and fisheries management) and have little room to expand routine sampling efforts to conduct periodic ecological baseline assessments, especially at state and local scales. Alternative design, monitoring, and assessment approaches are needed to help offset the burden of addressing additional data needs to increase understanding about estuarine system resilience when existing monitoring data are sparse or spatially limited. Research presented here offers a pseudo-probabilistic approach that allows for the use of found or secondary data, such as data on hand and other acquired data, to generate statistically robust characterizations of ecological conditions in estuaries. Our approach uses a generalized pseudo-probabilistic framework to synthesize data from different contributors to inform probabilistic-like baseline assessments. The methodology relies on simple geospatial techniques and existing tools (R package functions) developed for the US Environmental Protection Agency to support ecological monitoring and assessment programs like the National Coastal Condition Assessment. Using secondary estuarine water quality data collected in the Northwest Florida (US) estuaries, demonstrations suggest that the pseudo-probabilistic approach produces estuarine condition assessment results with reasonable statistical confidence, improved spatial representativeness, and value-added information. While the pseudo-probabilistic framework is not a substitute for fully evolved monitoring, it offers a scalable alternative to bridge the gap between limitations in resource management capability and optimal monitoring strategies to track ecological baselines in estuaries over time.

**Supplementary Information:**

The online version contains supplementary material available at 10.1007/s10661-024-12877-8.

## Introduction

Estuaries are transitional waters where freshwater from upstream and local watersheds mix with saltwater from the open ocean. From tidal rivers to the marine water interface, estuaries exhibit highly variable gradients of physio-chemical characteristics (e.g., salinity, dissolved oxygen), which contribute to their complexity, biological diversity and productivity, and natural resilience (Dame, 2008; Elliott & Quintino, 2007). Evidence suggests that estuaries may be at higher risk for ecological decline due to increasing anthropogenic pressure and the effects of climate change (Boucek et al., 2022; Cloern et al., 2016; Freeman et al., 2019; Lotze et al., 2006; Mahoney & Bishop, 2017; Pelletier et al., 2020). Long-term monitoring and periodic assessments play a critical role in developing effective estuarine management strategies. The collected data can produce meaningful information about ecological status and trends and highlight where improved environmental policies are needed (US EPA, 2008a). Such monitoring programs are implemented to provide snapshots regarding ecological function and conditions in estuaries across space and over time (Kennish, 2019; Paul et al., 1992). Despite recommendations to improve environmental status and trends monitoring (National Research Council, 1977; Ward et al., 1986), spatially comprehensive and comparable ecological data for estuaries remain relatively scarce (Gibbs, 2013; Shin et al., 2020) with a few notable exceptions such as the National Estuarine Research Reserves (https://coast.noaa.gov/nerrs/) and National Estuary Program (https://www.epa.gov/nep). Recent monitoring trends suggest that estuarine management programs are replacing long-term, spatially encompassing baseline monitoring with more place- or compliance-based monitoring (Carstensen et al., 2011; Gibbs, 2013). In the United States (US), state-level monitoring programs are under pressure to address numerous state and federal water quality priorities, such as setting total maximum daily load (TMDL) limits for primary pollutants and state-wide water quality assessments—in particular, requirements mandated under the US Clean Water Act (33 USC § 1251 et seq. as amended, 1972). Targeted, place-based monitoring is often a practical solution for accumulating the data needed to meet statutory reporting requirements amid budget and staffing fluctuations (Newmark & Witko, 2007). However, this shift in monitoring approaches tends to perpetuate existing gaps in estuarine ecology data, reducing our ability to recognize when a system is shifting toward a new ecological state (Elliott & Quintino, 2007; Pelletier et al., 2020) and new management strategies may be needed (Duarte et al., 2009; Elliott et al., 2019).

Increased monitoring without a robust design structure does not necessarily translate into better data to inform assessments. Statistical sampling frameworks, particularly probability-based, have been used to improve aquatic resource monitoring for decades (Blick et al., 1987; Carstensen, 2007; Olsen et al., 1997; Overton & Stehman, 1996; Stevens, 1994). In 2010, the US Environmental Protection Agency (EPA) implemented the National Coastal Condition Assessment (NCCA) (https://www.epa.gov/national-aquatic-resource-surveys/ncca) to establish a uniform monitoring approach to collect critical, nationally consistent information to answer fundamental questions about coastal water quality. The NCCA works in partnership with states and tribes to conduct field surveys once every 5 years to collect data in estuaries and nearshore areas of the Great Lakes. Based on data collected at relatively few randomly selected sampling locations, statistically robust, spatially balanced variance estimates can be made to characterize the extent to which certain ecological conditions exist at various spatial scales (Stevens & Olsen, 2003). Despite the statistical merits of using probabilistic monitoring and the success of the NCCA, most state-level estuarine data are collected through targeted or preferential sampling (Brus & de Gruijter, 2003), a practice that is prone to introduce sampling bias in reported results (Diaz-Ramos et al., 1996; Kermorvant et al., 2019;  Messer et al., 1991; Stevens & Olsen, 2003, 2004). With the growing worldwide concern regarding the resilience of coastal resources, more uniform, spatially representative, and consistent monitoring will be needed to successfully transition toward more adaptive and inclusive coastal resource management strategies (NOAA, 2023; United Nations, 2022).

The use of found or secondary data (e.g., data collected by others for distinctly unique objectives) is becoming an increasingly popular solution for addressing ecological data gaps, especially the integration of participatory science (Fraisl et al., 2022; Hampton et al., 2013; McKinley et al., 2017; Nelson, 2009; Thelen & Thiet, 2008; Tunnell et al., 2020). Combining data from different studies is not without issues (e.g., spatial autocorrelation, pseudo-replication, incongruent design assumptions), but data synthesis and standardization are more straightforward when all data are probabilistic (Maas-Hebner et al., 2015). However, many state and local programs in the US lack the capacity or the priorities to implement full-scale probabilistic sampling, and few appropriate alternatives are available (Elliott & Quintino, 2007). Earlier research explored the utility of simulating probabilistic designs using found data (Brus & de Gruijter, 2003; Overton et al., 1993). In both case studies, the authors concluded that approaches offered some limited utility given specific applications (e.g., streams and lakes), but the added value that the results offered was inconclusive. Currently, the use of spatial interpolation is a popular approach for preparing secondary data for subsequent analysis even though the literature suggests that spatial models are unreliable when applied to GIS representations of estuaries (Li & Heap, 2011; Little et al., 1997).

Readily transferable, statistically defensible approaches are still needed to assist states and local coastal programs in augmenting their ability to assess baseline ecological conditions in estuaries to better inform future resource management decision-making and priority setting. We offer a generalized, pseudo-probabilistic approach that leverages the strength of probability-based monitoring by mimicking hallmark features of the probability surveys implemented for the NCCA. This research offers an intermediate alternative for producing resource-wide estuarine assessments when the capacity to implement such field efforts is limited. In this study, all data were treated as secondary, regardless of site placement, to eliminate some of the complexities of integrating probabilistic and non-probabilistic data. A hexagonal grid was used to harmonize data to create discrete sampling units. A probabilistic design was integrated with derived sampling units to determine which data to include for an assessment and to help mitigate potential sampling bias that may have been inherited from acquired data. Using the publicly available “spsurvey” R package (Dumelle et al., 2023) helped to simplify the design development and data analysis processes. Here, we offer a detailed description of our approach and four use-case demonstrations using select water quality data collected in a subset of estuaries in northwest Florida. These demonstrations highlight the potential utility of our pseudo-probabilistic approach and its ability to characterize baseline ecological conditions in estuaries with statistical confidence.

## Methods

### Software

An estuarine GIS feature class was used to define the study area and serve as the spatial foundation of the pseudo-survey design and refinements were accomplished using ArcGIS Pro version 2.6.3 for Windows. Data analysis was completed using R for Windows version 4.1.3 (2022–03-10) (R Core Team, 2021) in the R-Studio IDE release 2022–01-04 (RStudio Team, 2021). The following lists the primary R-packages used to perform various data and geospatial processing and analytical functions in this study: data harmonization was performed using *dplyr* (v1.1.1; Wickham et al., 2023) and *tidyverse* (v2.0.0; Wickham et al., 2019) packages; *sf* package (v 1.0–12; Pebesma, 2018) was used for spatial data processing and manipulation; probabilistic sampling and testing were conducted using the *spsurvey* package (v5.4.1; Dumelle et al., 2022); and data visualizations using *ggplot2* (v3.4.2; Wickham, 2016) and *ggstatsplot* (v0.11.0; Patil, 2021). Other limited-use R-packages are listed in various methods sections.

### Study area

**Fig. 1:**
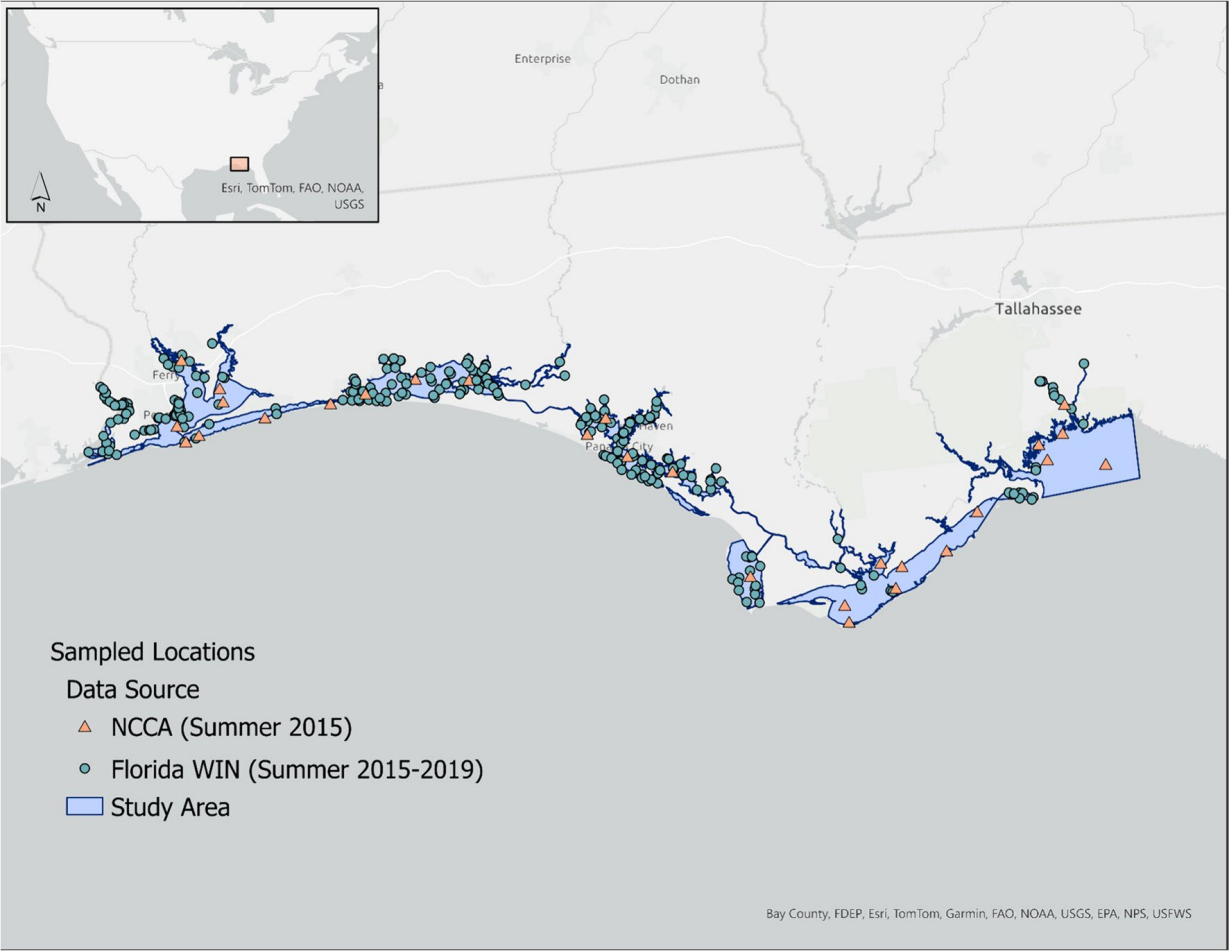
Map of the study area identifying locations where data, delineated by source, were collected during June–September 2015–2019 in the Northwest Florida Water Management District (NWFWMD)

The study area or target population encompassed all estuarine waters within the Northwest Florida Water Management District (NWFWMD). The resource extended from the northwest Florida panhandle at the Alabama-Florida state border to the mid-point of Apalachee Bay (Fig. 1). A GIS feature layer or sample frame was created to represent the target population and facilitate spatial analysis. The sample frame was extracted from an enhanced version of the United States Geological Survey’s 1:100,000 digital line graph (DLG). The resulting DLG enhancement was previously created to include all US marine tidally influenced waters and the adjacent nearshore open ocean (Bourgeois et al., 1998). The nearshore polygons within the sample frame provided the seaward boundary. Salinity zone polygons (Nelson, 2015) were added to identify the upstream boundary of each estuarine system. Finally, a GIS shapefile of NWFWMD county boundaries (“Northwest Florida Water Management District Boundaries with county divisions,” https://nwfwmd-open-data-nwfwmd.hub.arcgis.com/datasets/NWFWMD::nwfwmd-county-boundaries/about) was used to delineate the east–west extent of the study area. A straight line, extending from the shoreline to the outermost seaward polygon, was used to bisect a large open water feature (Apalachee Bay) managed by two different Florida water management districts. The sample frame for the study area encompassed a total estuarine area of 2705.90 km^2^.

### Data selection and evaluation

#### Data Sources

For this effort, surface water characteristic data were the primary focus. Salinity, temperature, pH, dissolved oxygen, chlorophyll *a*, total nitrogen, total phosphorus, and enterococci measures were retained as the suite of water quality parameters common among the contributing data sources. Surface water quality measurements (maximum depth 0.5 m) used in this study were collected from early to late summer (June–September) from 2015 to 2019, referred to as the index period. Figure 1 highlights the study area with the sampled locations (*n* = 345) delineated by the data source. Shapefile and metadata for the study area are available in supplementary material.

The data were retrieved from two publicly accessible sources: EPA’s NCCA website (“National Coastal Condition Assessment,” https://www.epa.gov/national-aquatic-resource-surveys/ncca) and FDEP’s Watershed Information Network (FWIN), a centralized environmental data management platform (“Welcome to Watershed Information Network WIN,” https://floridadep.gov/dear/watershed-services-program/content/winstoret). FWIN data were collected under different sampling designs, at varying frequencies, and by several divisions within the FDEP, Florida-based partner organizations, and participatory science groups (Table 1). Targeted sampling was the primary method used to acquire FWIN data although FDEP’s Water Quality Assessment Section uses a probabilistic design to collect status and trends data which may include small portions of tidal rivers. In some cases, FWIN sites were visited multiple times during the targeted index period, and parameters were not consistently collected during each site visit. For simplicity, the collection of Florida partner data is referred to as a single FWIN, non-probabilistic dataset. It represents the primary suite of available Florida secondary data used in the analysis. NCCA data were extracted from a national dataset collected in a coordinated fashion across US estuaries at locations determined using a true probabilistic design (Dumelle et al., 2023). All NCCA sites were visited once during the index period, and all parameter data were collected while field crews were on site. NCCA data were only available for 2015. In the context of this research, all data were treated as non-probabilistic. However, only water quality parameters common to both NCCA and FWIN were included in the analysis (Table 2). Data qualification, metadata, and other technical information, i.e., as referenced in FDEP (2022) and US EPA (2021), were used as screening tools to inform data selection. Two quality objectives governed the usability of secondary data for analysis purposes—accuracy and comparability.

**Table 1:**
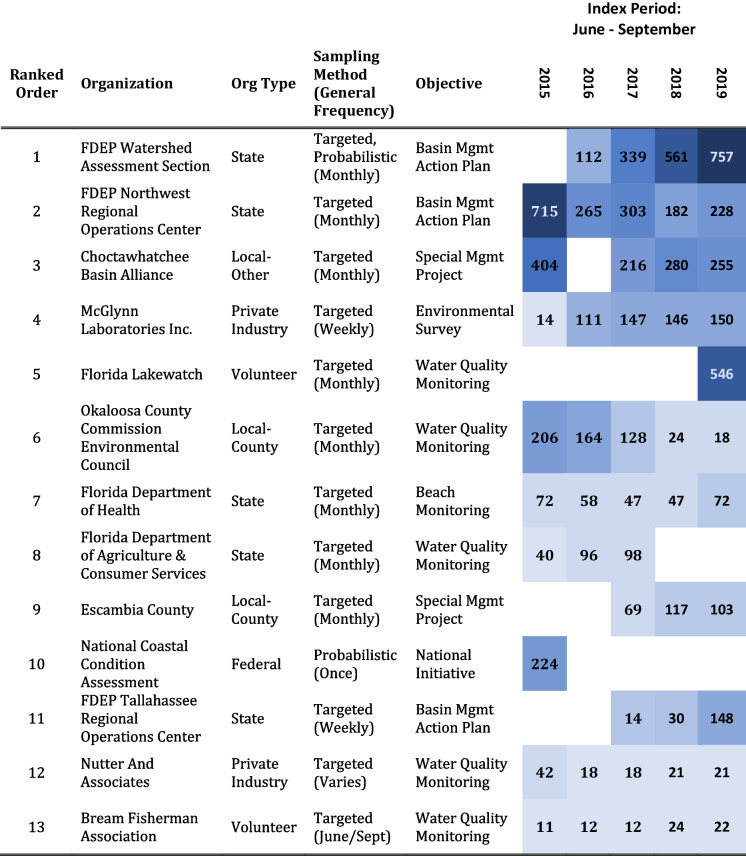
List of organizations that contributed data used in this study. Organizations presented in descending order (most significant to least) based on the number of unique surface-water data points contributed. Counts were summarized across all parameters of interest, by organization and year. The color gradient reflects each count relative to all counts in the matrix. Color becomes darker as values increase (no color = no data). Data collection objective, general site selection approach, and typical sampling frequency are also listed for each organization

**Table 2:**
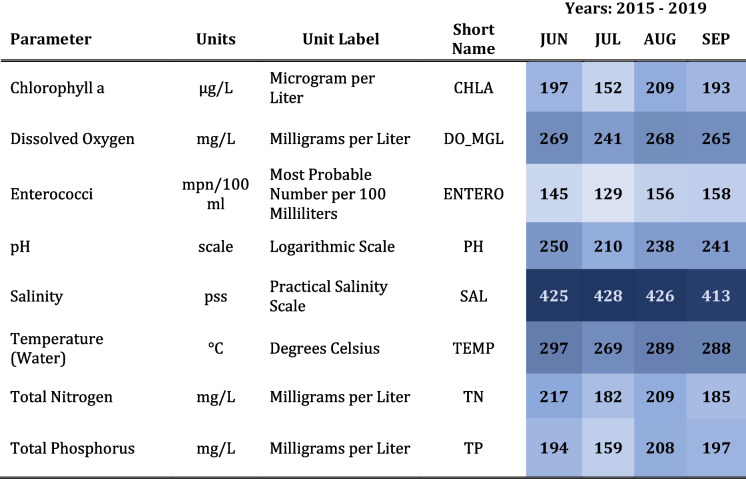
Selected water quality parameters used in demonstration assessments and the unit value to which each parameter was standardized. Parameters presented alphabetically. Counts were summarized by month (index period) and year. The color gradient reflects each count relative to all counts in the matrix. Color becomes darker as values increase (no color = no data)

#### Data accuracy

A review of published quality assurance plans for FWIN and NCCA programs was conducted to ensure that contributed data were qualified using rigorous methods (e.g., published literature and widely-accepted practices). For the FWIN data, two quality plans described the various sampling and laboratory operating procedures while a third outlined the quality control and governance of Florida’s Watershed Information Network (“DEP Quality Assurance Program for Sect. 106 Funded Activities”, https://floridadep.gov/dear/quality-assurance/content/dep-quality-assurance-program-section-106-funded-activities). One quality assurance plan was available for the NCCA data which included field and laboratory operations manuals describing field data collection and sample preparation and analytical methodologies (“Manuals Used in the National Aquatic Resource Surveys”, https://www.epa.gov/national-aquatic-resource-surveys/manuals-used-national-aquatic-resource-surveys#National%20Coastal%20Condition%20Assessment). While instrumentation and field equipment varied by contributing organizations (e.g., manufacturer, age, and calibration standards), there was a reasonable expectation that field and laboratory data were generally usable based on each programs stated data quality objectives and the extent to which data were said to have been reviewed. For this research, the use of existing data quality assessments for initial data acceptability screening seemed appropriate since FWIN and NCCA data are used to fulfill state and federal reporting requirements, respectively, under the US Clean Water Act (33 USC § 1251 et seq. as amended, 1972).

#### Data comparability

There were several data processing activities performed to ensure that data were adequately comparable for analysis. A range of months (June–September) was used to subset acquired data to help promote seasonal cohesion while maximizing data collection overlap between sources. Duplicate data and values reported without units or unit basis (e.g., per liter) were excluded as were data originally reported with exclusion qualifiers. The description of each qualifier code was reviewed, not only between different sets of data, but also over years to ensure that qualifiers were consistently interpreted. Data qualified with codes not expressly tagged for exclusion were assessed on a case by case basis to evaluate usability.

In cases where replicate measurements were present, such as water profile upcast/downcast readings, the mean value was calculated and retained for analysis. Parameter measures qualified as not detectable were set to the reported method detection limit if no other proxy value (e.g., practical quantitation limit) was provided. Units and unit basis (when appropriate) were standardized for each set of parameter data as described in Table 2. When necessary, specific conductance values were converted to salinity estimates (pss) based on equations published by Schemel (2001) as translated into an R function by Jassby and Cloern (2015). In some cases, dissolved oxygen units were converted from % saturation to mg/L using the “DO.unit.convert” function of the R *rMR* package (Moulton, 2018) based on methods published by Benson and Krause (1984).

Since estuarine water quality measurements can naturally fluctuate between extremes, it was important to address potential outliers in a uniform manner. Using quartiles, data were summarized within each parameter group to examine distributions for anomalies. Data values falling beyond three interquartile ranges from the 1st and 3rd quartiles were reviewed as potential outliers. Average monthly precipitation data from the Florida State University Climate Center (https://climatecenter.fsu.edu/products-services/data/statewide-averages/precipitation) and site placement within the waterbody (e.g., open bay and tributary) were used to determine if sites with low salinity values (salinity ≤ 0.05 pss) should be dropped from the study because they were outside the area of interest (in freshwater). Previously reported water quality measurements using data collected in 1991–1994 and 2000–2006 (US EPA, 2001, 2004, 2008b, 2012) were used to help distinguish potential outliers from erroneous entries. Outlier data were kept if there was no evidence to suggest that extreme values were a product of an entry error (e.g., misplaced decimal point). For demonstration purposes, a 99% Winsorization (Tukey, 1962) was applied to data within each parameter group to minimize the analytical impact of extreme upper and lower values and still capture the typical variability associated with estuarine water quality characteristics.

### Simulating a probabilistic survey design

#### Synthesis of secondary data

**Fig. 2:**
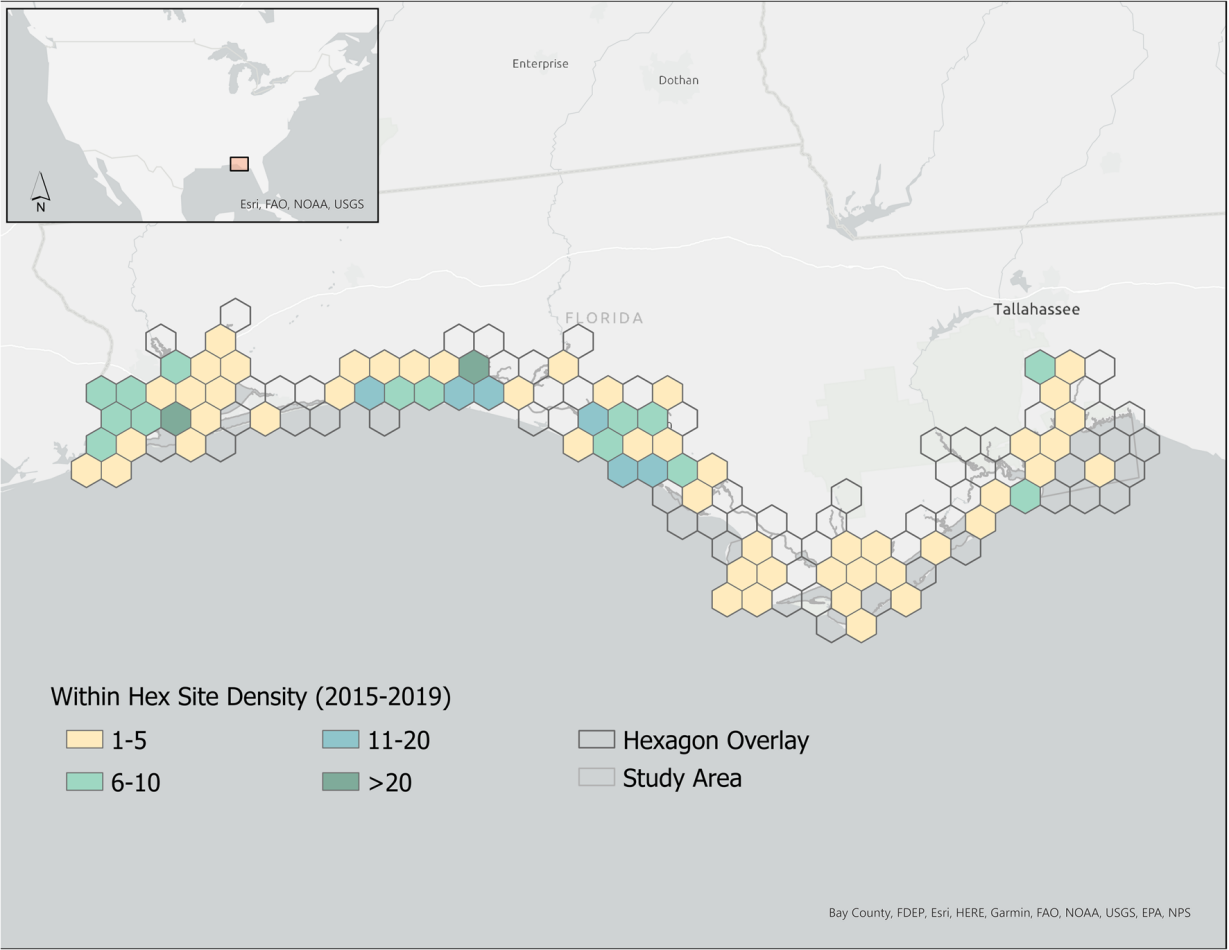
Map depicting the hexagonal grid overlay with cells highlighted by the number of sampled sites (2015–2019) contained within each hexagon. Hexagons without color = no sites

All data were processed in the same manner. Collectively, data were harmonized to address variations in site selection methods (designs), parameter collection, and sampling frequency. Using the “st_make_grid” function in the R *Simple Features* package (Pebesma, 2018), sampling *units* were derived using a hexagon overlay to summarize parameter data encompassed within a cell to produce a suite of “composite” sites for each year of available data. To simplify and standardize the process, the size of each hexagon used to summarize data was determined by area, calculated as the total study area (km^2^) divided by the number of sites (*n* = 45) chosen to characterize water quality. The number of sites reflects the minimum number of observations needed for a statistically robust probability analysis (30) plus a pad of fifteen (1.5 ×) to account for the random selection process described in the “Pseudo-probabilistic design—part 1” and “Pseudo-probabilistic design—part 2” sections. Only hexagons intersecting with the study area were retained to create a resource-fitted grid overlay (*n* = 134 hexagons). To account for temporal and sampling frequency differences, data within each hexagon were summarized by parameter and year using the geometric mean. The mean longitude and latitude of sampled sites represented the composite location (x,y-coordinate where samples were taken within a hexagon. Each composite site was assigned a unique hexagon identifier to facilitate association with design information generated in the methods outlined in the following sections. The distance between hexagon center points was approximately 10 miles (16 km). The suite of composited data was used as the basis for each demonstration assessment. Figure 2 shows the distribution and density of sampled sites co-located within each hexagon where data existed. Details and composited data are shown in supplementary material.

#### Pseudo-probabilistic design—part 1

Using a generalized random tessellation stratified (GRTS) survey design, 90% confidence in population variance estimates can be achieved with a minimum of 30 randomly generated, spatially balanced sites (Stevens & Olsen, 2003). To that end, an authentic probabilistic survey design was produced as the foundation of the pseudo-probabilistic framework. GRTS is a tool, available through the R *spsurvey* package (Dumelle et al., 2022), that simplifies the process of creating spatially balanced, statistically robust survey designs to support environmental monitoring. In brief, the GRTS code library generates a densely populated grid of uniformly spaced x,y-coordinates placed in a GIS-rendered proxy of a natural resource, such as estuaries (Stevens & Olsen, 2003; Theobald et al., 2007). A recursive tessellation technique extracts a random subset of coordinates to produce a list of design or intended sampling locations (Stevens & Olsen, 2003).

GRTS supports the development of complex survey design configurations (Dumelle et al., 2023). However, for this study, we employed a simple, unstratified design. Only the number of sites (*n* = 45) was supplied to initiate GRTS, with default code parameters accepted for all other function parameters. The resource grid overlay used to summarize raw data was merged with the GRTS-generated sites to capture design information used to produce assessment estimates (e.g., inclusion probability). In some instances, differences between the two hexagon constructs placed more than one design site within a single data hexagon (see supplementary material for more detail). To resolve this problem, only the first GRTS site chronologically generated within a data hexagon was retained. The final pseudo-probabilistic design framework was comprised of 36 unique sites.

#### Pseudo-probabilistic design—part 2

**Fig. 3:**
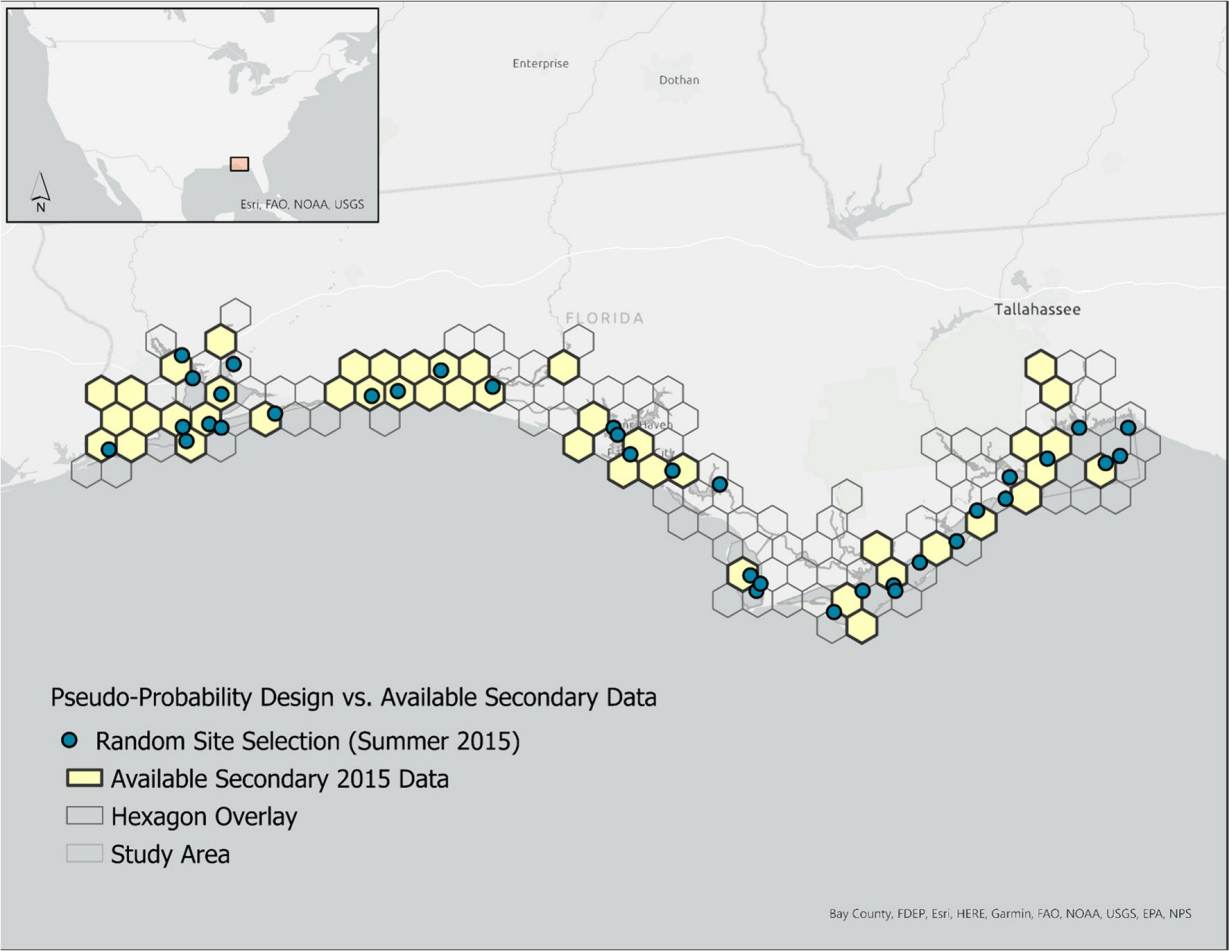
For clarity, the map shows the components of the synthetic probability design used to produce a single year (2015) pseudo-probabilistic assessment. The randomly selected design sites (*n* = 36 dots) were GRTS generated independently from areas known to have data (yellow hexagons). Dots represent GRTS-produced randomly selected sites. Dots found outside the yellow hexagons indicate areas where data were not available but retained for analysis purposes. Yellow hexagons (composite site) without a dot were excluded. An interactive version of this map is available in supplementary material

The steps discussed in this section were executed each time an assessment was produced since the actual population of data differed between scenarios. Composite sites were assimilated into the pseudo-probabilistic design framework using the hexagon identifier for reconciliation. A subset of data was extracted from the full complement of composited data to inform specific assessments based on the information available in the synthesized design. An example illustration (Fig. 3) shows GRTS-generated sites in the context of where composite site data are located for the 2015 assessment demonstration. All randomly selected hexagons were retained for analysis purposes. Hexagons that did not pair up with a GRTS-generated site were not included in any subsequent assessments. A weighting factor assigned to each site is needed to calculate probability estimates. For demonstrations, all sites were given equal weighting, calculated as the total estuarine area (km^2^) of the study area divided by the number of sites included in a specific scenario.

### Pseudo-probabilistic assessments

**Fig. 4:**
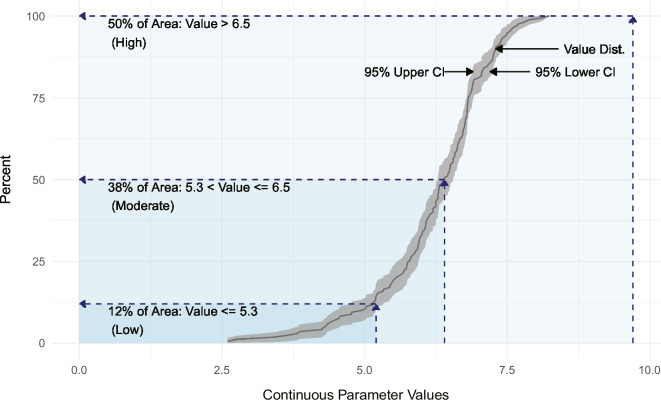
Illustration showing the use of CDF to produce categorical estimates using synthetic data. Percent of estuarine area estimates (y-axis) are determined relative the threshold values delineated along the x-axis. Estimates are calculated by subtracting the maximum area value of previous category from maximum area value of category of interest

The *spsurvey* package uses the cumulative distribution function (CDF) with 95% confidence intervals as the primary tool to produce assessment estimates based on the distribution of observed assessment data and calculated variance estimates (Stevens & Olsen, 2003). An assessment result describes the estimated percent of estuarine area exhibiting some defined ecological characteristic (e.g., concentrations of TN, water quality) with known confidence. Assessment estimates are determined based on the intersection of the x- and y-axis values along the CDF curve, where “x” represents the range of measured parameter values and “y” shows the percentiles of the distribution of a population of x-values. In this case, the target population is 100% of the estuarine area in the study’s boundaries. Confidence intervals are calculated using the Horvitz-Thompson variance estimation method in the context of a continuous resource survey designs (Stevens, 1997; Stevens & Olsen, 2003) (Fig. 4).

Threshold values were applied as “breaks” on the x-axis of the CDF, which was composed of the continuous measurements of a parameter. These breaks represented the demarcation points used to assign categorical attributes for each parameter set. For this study, threshold values (Table 3) were derived from quartiles and the Florida Surface Water Quality Standards (rev. 2017). For 1-year and 5-year assessments, “Low” was assigned to data values falling below the 25th quartile for a given parameter. Data values exceeding the 75th quartile were assigned a “High” category while the remainder of data was set to “Moderate.” Florida’s published standards for estuarine resources are specific for each estuary. For simplicity, the Florida 3-year assessments excluded nutrient and NCCA data. The mean values of the reported parameter criteria that were relevant to the waterbodies within the study area served as the benchmark for assigning either an “Above” or “At/Below” category to the Florida-specific parameter data. In all cases, “Not sampled” was applied as the category for missing parameters.

**Table 3:** Threshold values used to inform categorical assessments. Quartiles were established using raw FWIN and NCCA data collectively

Parameter	Quartile-based		Florida criteria based
	25th	75th	
Chlorophyll a (µg/L)	1.80	6.55	5.44
Dissolved oxygen (mg/L)	5.88	7.09	5.54
Enterococci (MPN/100 ml)	5	20	35
pH (scale)	7.6	8.1	6.5
Salinity (pss)	0.1	21.1	0.5
Water temperature (°C)	28.7	31.0	28.3
Total nitrogen (mg/L)	0.305	0.492	NA
Total phosphorus (mg/L)	0.013	0.027	NA

To produce assessments, the percent area values were generated by subtracting the maximum percent area related to the previous category grouping (or zero) from the maximum area encompassed in the category of interest. The y-axis denoted the full extent of the study area (0 to 100%).

The “cat_analysis” *{spsurvey}* function produced assessment results reflecting the percent of estuarine area that fell within each category grouping by parameter. Spatial balance, precision, and population differences were analyzed using the “sp_balance,” “cont_analysis,” and “change_analysis” *{spsurvey}* functions, respectively. Each assessment was based on a minimum of 30 sites derived from the pseudo-probabilistic design. Variance estimates representing the percent of estuarine area characterized by assessment category (e.g., low, moderate, and high) were calculated based on the population estimation methods described in Stevens and Olsen (2003, 2004).

## Results

### Descriptive statistics

Based on longitude and latitude, 345 unique raw data locations were sampled during the 5-year index period (2015–2019). The frequency of visits per site ranged from 1 to 10 during the same time span. Descriptive summaries of the resulting raw secondary dataset are presented in Table 4.

**Table 4:** Distribution characteristics of the full complement of Florida FWIN and NCCA raw water quality secondary data points for June–September 2015–2019

Assessment Period: 2015	Florida WIN (Summer 2015–2019 data points)						NCCA (Summer 2015 data points)					
Parameter	*n*	Quartiles			Mean	Std Dev	*n*	Quartiles			Mean	Std Dev
		25th	50th	75th				25th	50th	75th		
Chlorophyll *a* (µg/L)	723	1.8	3.4	6.7	5.4	5.6	28	2.3	2.8	4.1	4.4	5.0
Dissolved oxygen (mg/L)	1015	5.8	6.6	7.1	6.4	1.2	28	6.1	6.7	7.1	6.6	0.9
Enterococci (MPN/100 ml)	560	5.0	5.0	22.0	45.0	120.3	28	0.0	0.0	0.0	34.8	121.2
pH (scale)	1664	7.5	7.9	8.1	7.8	0.5	28	7.9	8.0	8.1	8.0	0.2
Salinity (pss)	1115	0.1	10.6	20.7	11.6	10.8	28	17.1	25.9	29.6	22.6	9.7
Water temperature (°C)	765	28.6	29.9	31.0	29.7	2.0	28	29.1	30.6	31.0	30.2	1.5
Total nitrogen (mg/L)	730	0.307	0.380	0.498	0.414	0.167	28	0.294	0.331	0.378	0.360	0.102
Total phosphorus (mg/L)	911	0.013	0.017	0.027	0.022	0.014	28	0.014	0.015	0.023	0.019	0.010

During the data harmonization process, it was found that approximately 1/3 of the hexagons included sites that would likely be spatially autocorrelated if considered independently, a consequence of targeted site selection (Brus & de Gruijter, 2003).

Wilcoxon rank sum with continuity correction or Mann–Whitney *U* analyses were performed to identify differences in the distribution of parameter values between NCCA and FWIN secondary data (Table 5). Results including only 1-year (2015) of data suggested that the distribution for chlorophyll *a* (CHLA), enterococci (ENTERO), salinity (SAL), water temperature (TEMP), and total nitrogen (TN) observations differed significantly between the two datasets. However, when comparing the 2015 NCCA to 5 years of FWIN data, the difference in distribution profiles for CHLA, TEMP, and TN disappeared. There was no significant difference in distributions for dissolved oxygen (DO_MGL) or total phosphorus (TP) when comparing 2015 alone and 2015–2019. There was a significant difference in the distribution ENTERO between NCCA and 5 years of raw data for FWIN.

**Table 5:** Results for 2015 and 2015–2019 Wilcoxon rank sum test with continuity correction (Mann–Whitney *U*) comparing the raw 2015 NCCA data with raw FWIN data from 2015 or FWIN data collected between 2015 and 2019. Box plot data distributions are available in the supplementary material

Parameter	Mann–Whitney *U*			
	2015		2015–2019	
	W_Mann-Whitney_ statistic	< *p* >	W_Mann-Whitney_ statistic	< *p* >
Chlorophyll *a* (µg/L)	**1096.5**	**0.05**	10672.5	0.63
Dissolved oxygen (mg/L)	2873.5	0.22	13166.5	0.51
Enterococci (mpn/100 ml)	**4214.0**	** < 0.001**	**14072.0**	** < 0.001**
pH (scale)	2956.0	0.51	**9201.2**	**0.01**
Salinity (pss)	**2903.5**	** < 0.001**	**9985.0**	** < 0.001**
Water temperature (°C)	**3518.5**	**0.04**	13091.0	0.14
Total nitrogen (mg/L)	**1318.5**	** < 0.001**	12876.0	0.07
Total phosphorus (mg/L)	1772.0	0.14	10625.0	0.72

### Spatial representativeness

Pielou’s Evenness analysis was conducted using the “sp_balance” {spsurvey} function to evaluate the spatial balance of sampled locations pre- and post-application of the pseudo-probabilistic design. Pielou’s Evenness Index (PEI) ranges from 0 to 1. In the context of probability designs, spatial balance improves as the index moves closer to 0 (Dumelle et al., 2023). Table 6 shows the PEIs for raw secondary data for 2015 and 2015–2019, both with and without the NCCA 2015 contribution, and NCCA data before applying the pseudo-probabilistic site selection. A single pseudo-probabilistic design was applied to all datasets to facilitate assessments. The post-design application measurement for NCCA was “NA” because NCCA data were not assessed independently. The NCCA sites were more spatially balanced than any other evaluation using FWIN sites (evenness = 0.20 to 0.35). Secondary data exhibited spatial balance characteristics similar to that seen in the probabilistic 2015 NCCA data after applying the pseudo-probabilistic design.

**Table 6:** Results from Pielou’s Evenness analysis showed an improvement in spatial representation after applying the pseudo-probabilistic framework. Spatial balance is greater as the index moves closer to 0.00

Spatial Balance Assessment (Pielou’s Evenness Index)		
	Pre-framework application	Post-framework application
FWIN 2015	0.35	0.04
FWIN 2015–2019	0.25	
Combined FWIN 2015 + NCCA 2015	0.21	
Combined FWIN 2015–2019 + NCCA 2015	0.20	
NCCA 2015	0.07	NA

### Data representativeness

Null hypothesis simulations were conducted to examine the potential combined effects of the hexagon-assisted data summarization (composited parameter data) and the application of the pseudo-probability selection process on assessment data representativeness. For each parameter, random distribution simulations were generated using the 2015 and 2015–2019 datasets containing combined FWIN + NCCA pseudo-probabilistic data to see how often the simulated mean fell within the 95% CI of the true mean of observed data. After 1000 simulations, results (*p*-value) indicated that the pseudo-probabilistically selected data reasonably replicated the distribution of observed response data (Table 7). Results were similar for the 2015 and 2015–2019 simulations.

**Table 7:** Comparisons between mean raw data values and 1000 distribution simulations using random subsets of the pseudo-probabilistically selected data created from the same raw data observations

Parameter	2015		2015–2019	
	Observed mean ± Std Dev (# observations)	Null-hypothesis estimated mean ± Std Dev *p*-value	Mean ± Std Dev (# observations)	Null-hypothesis estimated mean ± Std Dev *p*-value
Chlorophyll *a* (µg/L)	5.8 ± 5.7 (*n* = 90)	6.03 ± 5.55 *p* = 0.92	5.3 ± 5.6 (*n* = 751)	4.91 ± 3.38 *p* = 0.98
Dissolved oxygen (mg/L)	6.2 ± 1.4 (*n* = 267)	5.58 ± 0.8 *p* = 0.99	6.4 ± 1.2 (*n* = 1043)	6.39 ± 0.93 *p* = 0.98
Enterococci (MPN/100 mL)	35.3 ± 103.9 (*n* = 196)	7.06 ± 2.86 *p* = 0.99	44.5 ± 120.3 (*n* = 588)	19.97 ± 51.93 *p* = 0.88
pH (scale)	8.0 ± 0.5 (*n* = 224)	7.97 ± 0.22 *p* = 0.91	7.8 ± 0.5 (*n* = 939)	7.8 ± 0.42 *p* = 0.99
Salinity (pss)	17.9 ± 9.0 (*n* = 367)	18.53 ± 7.41 *p* = 0.97	11.79 ± 10.9 (*n* = 1692)	16.67 ± 8.75 *p* = 0.98
Water temperature (°C)	29.4 ± 2.1 (*n* = 357)	29.35 ± 0.43 *p* = 0.99	29.7 ± 2.0 (*n* = 1143)	29.6 ± 1.21 *p* = 0.99
Total nitrogen (mg/L)	0.433 ± 0.139 (*n* = 92)	0.420 ± 0.10 *p* = 0.95	0.412 ± 0.165 (*n* = 793)	0.406 ± 0.125 *p* = 0.99
Total phosphorus (mg/L)	0.022 ± 0.013 (*n* = 135)	0.020 ± 0.010 *p* = 0.95	0.021 ± 0.013 (*n* = 758)	0.02 ± 0.009 *p* = 0.93

### Demonstrations

The same pseudo-probabilistic framework (*n* = 36 design sites) was used to determine which composite sites to include in each assessment. The number of sites supporting each assessment varied based on the years associated with the scenario. Assessments were produced using the “cat_analysis” function *{spsurvey}* except for the change analysis. Side-by-side assessment results were generated using FWIN only and combined FWIN and NCCA data. Additional R-packages or functions are introduced in respective sections below, as appropriate. Assessment results are available as tables in the supplementary material.

#### Single-year assessment

**Fig. 5:**
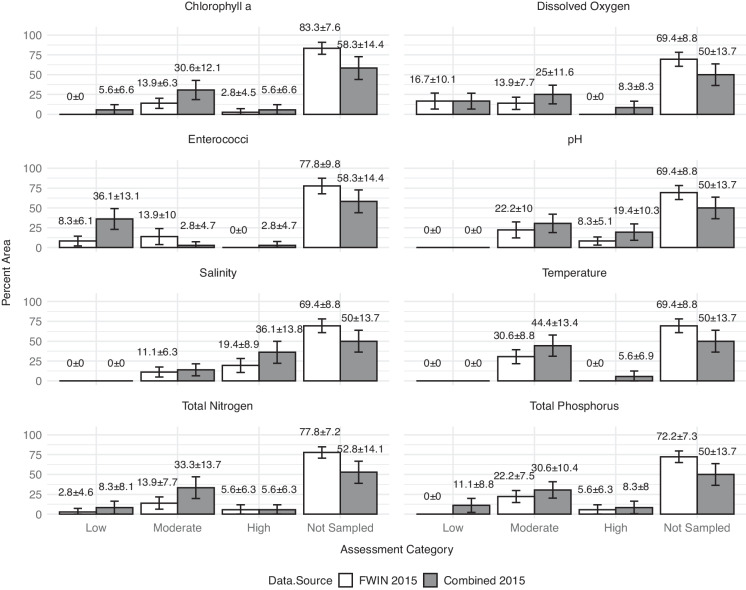
Illustration of 2015 assessments using the pseudo-probabilistic data showing the percent of estuarine area corresponding to quartiles of the combined NCCA and FWIN raw data (< 25th quartile = “low,” 25th–75th quartiles = “moderate,” > 75th quartile = “high”) water quality condition categories (Table 3) using 2015 FWIN and combined FWIN + NCCA-derived datasets

Pseudo-probabilistic data for the 2015 survey period were the basis for the 1-year assessment (Fig. 5). The application of the pseudo-probabilistic design to available secondary data identified 36 sites each for the FWIN only and FWIN + NCCA assessments. Pseudo-probabilistic data values were compared to quartiles (Table 3) from each parameter’s combined NCCA and FWIN raw data. The most notable benefit attributed to the application of the framework was the significant decrease in the estimates for “Not Sampled” estuarine areas in the CHLA, TN, and TP assessments (based on calculated margin of error, MOE). For these three parameters, the increase in the area assessed (resulting from adding the 2015 NCCA data) appeared to have had a greater, but not significant, redistribution influence on estimates in the “Moderate” category. The difference between FWIN 2015 only and combined 2015 data estimates were significant for “Low” and “Moderate” ENTERO assessment. The MOE was greater for combined FWIN + NCCA 2015 ENTERO estimates than for FWIN 2015 only estimates.

#### Combined 2015–2019 Assessment

**Fig. 6:**
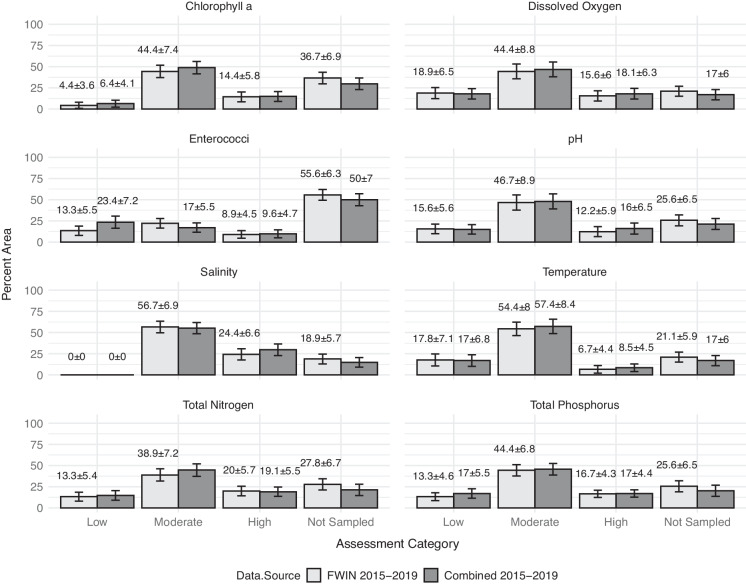
Illustration of 5-year assessments (2015–2019) showing the percent of estuarine area that met quartile based water quality condition categories ("low", "moderate", "high") (Table 3) using FWIN and combined FWIN + NCCA-derived datasets

Figure 6 shows assessment results using 5 years of pseudo-probabilistic FWIN (*n* = 90) and FWIN + NCCA (*n* = 94) data. This subset of data was also compared to quartile thresholds to produce probability estimates depicting the percent of estuarine area that fell within each category by parameter. In this demonstration, the addition of NCCA data had less influence on improving the spatial representation of the assessments. There were no instances of significant differences between the two assessment estimates.

#### Florida water quality standard-based assessments

**Fig. 7:**
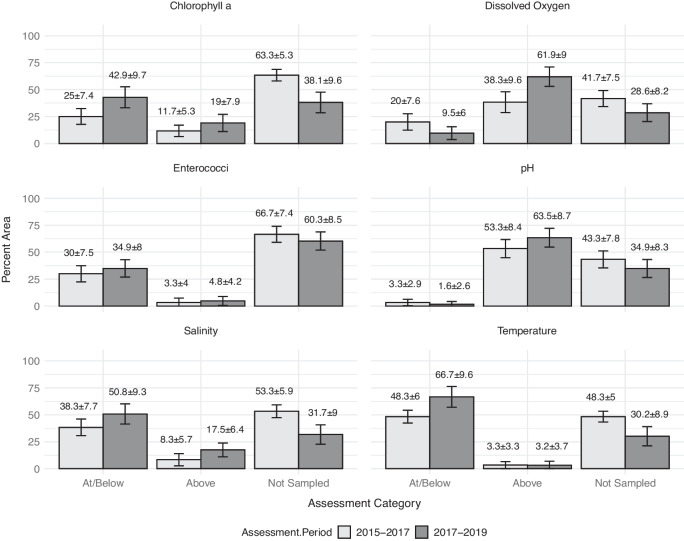
Illustration of two 3-year assessments (2015–2017 and 2017–2019) showing the percent of estuarine area that fell within water quality condition categories based on Florida Water Quality Standard Thresholds (Table 3) using FWIN suppled data only

FDEP does not include estuaries or marine coastal resources in their status or trend monitoring networks. However, FDEP reports on the “Designated Use” of estuarine resources every 2 years (FDEP, 2020, 2022). The FDEP assessment methodology could not be directly replicated. Instead, a pseudo-probabilistic adaption for the third demonstration was created (Fig. 7). Based on Florida’s reported methods, the assessment comprised two periods spanning 3 years each, 2015–2017 (*n* = 60) and 2017–2019 (*n* = 63). For this demonstration, two distinctly different subsets of data were created to represent the reporting periods. Data collected in June and July 2017 were kept for the 2015–2017 assessment, while data collected in August and September 2017 were included with the 2017–2019 assessment. Only FWIN data were included for this demonstration. Pseudo-probabilistic data were categorized into three categories: “At/Below” threshold, “Above” threshold, or “Not Sampled.” A significant reduction in the area “Not Sampled” was observed (based on MOE) in the 2017–2019 reporting period, along with a concomitant increase in the “At/Below” category for all three parameters. Additionally, there was a significant increase in the area exceeding the Florida-based threshold value for DO (“Above” category).

#### Change analysis

**Fig. 8:**
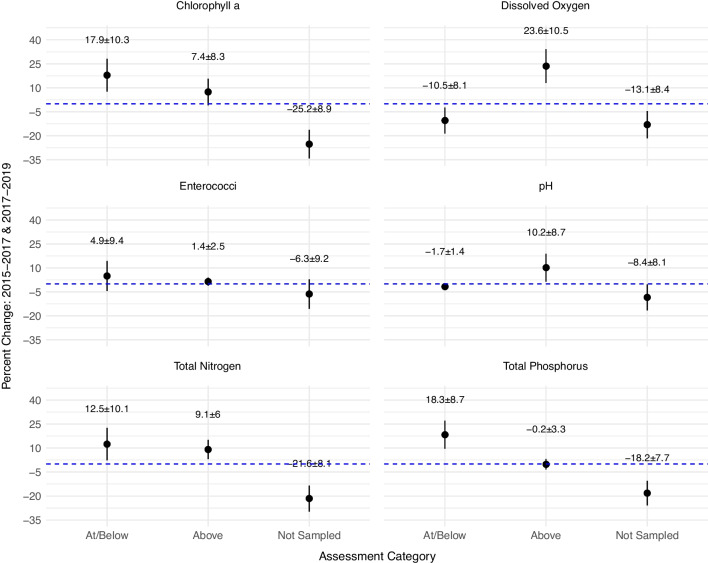
Illustration depicting the percent change in estuarine area exhibiting parameter characteristics indicative of each category highlighted in Fig. 7, for two different time periods: 2015–2017 and 2017–2019. Dots represent the change estimates and vertical line shows the 95% confidence intervals. Includes FWIN supplied data only. Blue dashed line identifies the zero (0) mark on the y-axis

Several functions within the “spsurvey” package offer options to examine estimates in different contexts (e.g., relative risk to ecotoxicological exposure). Figure 8 demonstrates the change analysis feature (“change_analysis” *{spsurvey}*). Using assessment results described in the “Florida water quality standard-based assessments” section, appreciable differences in assessment results can be calculated between the 2015–2017 and 2017–2018 reporting periods. Results are interpreted as estimates of the percent change in the extent of the resource area exhibiting water quality conditions that met or exceeded Florida-based thresholds. As expected, the change analysis results followed the same patterns observed in the previous analysis, where significant decreases were observed in the “Not Sampled” category for CHLA, TN, and TP and a significant increase in the “Above” category for DO.

### Performance of the pseudo-probabilistic estimates

Estimated mean values for each parameter were calculated using the continuous estimation methodology in the “cont_analysis” *{spsurvey}* function and compared with actual population means from secondary data observations (Table 8). Estimated means were derived from cumulative distribution functions calculated for the pseudo-probabilistic data distribution estimates. Measurement error is based on the 95% confidence interval. Overall, there was good correspondence between the raw data and pseudo-probabilistic estimates, which had low error. The root mean square error (RMSE) was calculated using the “sp_balance” *{spsurvey}* to evaluate how well the pseudo-probabilistic design represented the study area. In this case, the randomly sampled sites used in the pseudo-probabilistic design performed well (*p* < 0.05) in representing the study area in demonstration assessments.

**Table 8:** Comparisons between actual observed mean values of secondary data (± standard deviation) and pseudo-probabilistically estimated mean values (± measurement of error) of combined FWIN and NCCA data for 2015 and 2015–2019. The root mean square error shows how well the pseudo-probabilistic design (PP) represents the study area

Parameter	FWIN + NCCA 2015			FWIN + NCCA 2015–2019		
	Mean ± Std Dev (# observations)	PP estimated mean ± MOE (# observations)	PP spatial model RMSE	Mean ± Std Dev (# observations)	PP estimated mean ± MOE (# observations)	PP spatial model RMSE
Chlorophyll *a* (µg/L)	5.8 ± 5.7 (*n* = 90)	4.4 ± 2.0 (*n* = 15)	0.015	5.3 ± 5.6 (*n* = 751)	4.91 ± 0.6 (*n* = 66)	0.016
Dissolved oxygen (mg/L)	6.2 ± 1.4 (*n* = 267)	6.2 ± 0.3 (*n* = 18)		6.4 ± 1.2 (*n* = 1043)	6.4 ± 0.2 (*n* = 78)	
Enterococci (MPN/100 ml)	35.3 ± 103.9 (*n* = 196)	14.9 ± 22.6 (*n* = 15)		44.5 ± 120.3 (*n* = 588)	20.0 ± 12.9 (*n* = 47)	
pH (scale)	8.0 ± 0.5 (*n* = 224)	8.0 ± 0.1 (*n* = 18)		7.8 ± 0.5 (*n* = 939)	7.8 ± 0.1 (*n* = 74)	
Salinity (pss)	17.9 ± 9.0 (*n* = 367)	21.7 ± 2.7 (*n* = 18)		11.79 ± 10.9 (*n* = 1692)	16.7 ± 1.0 (*n* = 80)	
Water temperature (°C)	29.4 ± 2.1 (*n* = 357)	29.8 ± 0.4 (*n* = 18)		29.7 ± 2.0 (*n* = 1143)	29.6 ± 0.2 (*n* = 78)	
Total nitrogen (mg/L)	0.433 ± 0.139 (*n* = 92)	0.367 ± 0.036 (*n* = 17)		0.412 ± 0.165 (*n* = 793)	0.406 ± 0.018 (*n* = 74)	
Total phosphorus (mg/L)	0.022 ± 0.013 (*n* = 135)	0.019 ± 0.004 (*n* = 18)		0.021 ± 0.013 (*n* = 758)	0.020 ± 0.001 (*n* = 75)	

## Discussion

Currently, much of the available estuarine data is collected using targeted rather than random sampling, which can limit our ability to identify data patterns that signal when conditions in estuarine ecosystems may be changing and more adaptive management strategies may be warranted (Elliott & Quintino, 2007; Pelletier et al., 2020). The comparison of the secondary datasets used in this study (FWIN 2015–2019 and NCCA 2015) indicated that many parameters exhibited different data distribution profiles for the 2015 and 2015–2019 periods. This finding suggests that the two datasets likely represented different population samples, and drawing inferences from analysis conducted using only a single source could be misleading. While we were able to assess distribution differences, the Mann–Whitney test cannot reasonably detect sampling bias, a persistent challenge to overcome when using secondary data in ecological studies (Li & Heap, 2011; Little et al., 1997; Maas-Hebner et al., 2015).

The secondary data was evaluated for spatial balance, a determinate of sampling bias, using the software described by Dumelle et al. (2023). As expected, the NCCA sites were more spatially balanced than the FWIN sites. However, the lack of spatial improvement when NCCA and FWIN data were combined showed little effect, suggesting that the much larger volume of FWIN non-probabilistic data might overwhelm any spatial distribution benefit gained from including the NCCA probabilistic data. After secondary data were summarized (composite site data) and the pseudo-probabilistic design was applied, the composite sites exhibited spatial balance similar to the NCCA data. These results indicate that sampling bias was reduced using the pseudo-probabilistic approach.

A hexagon overlay was used to harmonize all data, irrespective of probability status. While including secondary probabilistic data likely improves spatial representativeness, the data are still being used outside the context of the original sampling objectives. To properly use found probabilistic data as a catalyst in ecological studies requires a statistically rigorous approach, such as the methods published by Overton et al. (1993), when augmenting with non-probabilistic data. With that in mind, all data were treated as non-probabilistic to simplify the data synthesis process. GIS polygon overlays (e.g., squares and triangles) used to partition an area into discrete and scalable spatial units are well established in the literature (Bousquin, 2021; Sahr et al., 2003; Stevens, 1997; Theobald et al., 2007; White et al., 1992). Hexagons introduce the least amount of spatial distortion when partitioning irregular GIS polygons and provide a uniform base for summarizing data. For this study, the number of sites rather than the length of a hexagon side (traditional method) determined the size of each hexagon in the resource grid used to summarize secondary data. By focusing on the number of sites, the approach for setting hexagon size was not only more intuitive, but also a practical way to standardize the process for use in different scenarios and spatial scales. The need to summarize data is apparent as shown in the heat map feature in Table 1 which highlights the inconsistency observed in sampling across the years in terms of frequency. In this study, the hexagon-assisted data aggregation helped maintain much of the variability expected in estuarine ecology data while minimizing the effects of sampling design differences across data sources (e.g., sampling frequency and location selection). Characterizing ecological conditions based on polygon aggregated data is becoming increasingly popular in ecological research (Birch et al., 2007; Bousquin, 2021).

Incorporating GRTS as part of the pseudo-probabilistic design strategy helped to ensure that the significant reduction in sampled locations would still provide statistically reliable assessment estimates. The purpose was to introduce the design characteristics intrinsic to probabilistic ecological assessments: randomness and spatial balance. There was a concern that the process used to harmonize observed data combined with the pseudo-randomized site selection would produce a distribution of data values that deviated substantially from the original data. However, null distribution simulations indicated that the pseudo-probabilistic site selection processes had little impact on the comparability between the observed and pseudo-probabilistic data, even though the data available in the latter dataset was significantly reduced. The pseudo-probabilistic design’s unstratified, equal probability feature may have artificially inflated simulated distribution probabilities. The GRTS selection process generates sites proportionally by discrete resource area polygons. This study summarized the sample frame into a single, unstratified polygon to facilitate site placement neutrality. Consequently, a larger percentage of randomly selected locations were placed in open-water areas where water quality characteristics tend to be more consistent (Fig. 3). A stratified design construct might have increased the spatial representation of shoreline/upstream areas, thus introducing more variability in the random sample (Stevens, 1997).

The assessment demonstrations were designed to highlight the flexibility of this pseudo-probabilistic approach for conducting secondary data ecological assessments. Equally important, the scenarios quantified the spatial limitations of data available for assessments, identified as the percent of resource area “Not Sampled.” Adding a second source of data (NCCA) to the FWIN data significantly reduced the amount of unassessed area for the single-year (2015) demonstration in some cases. As a result, there were notable differences in the redistribution of area estimates across assessment categories, i.e., “low,” “moderate,” and “high.” These results offered further evidence that FWIN and NCCA observation data were likely collected from different sample populations. Conclusions drawn strictly from a single source of secondary estuarine data (e.g., FWIN-only) could be skewed due to sampling bias (targeted placement of sampled sites).

On the other hand, the probabilistic influence of the NCCA data diminished significantly in the 5-year assessment (2015–2019), indicating that sampling frequency may play an important role when collecting secondary data for assessing ecological baselines over time. These 5-year assessment results agree with previous findings suggesting that the probabilistic nature of NCCA data did little to mitigate the spatial imbalance of the full complement of FWIN data when combined into a single dataset. Comparable sampling frequency of secondary estuarine data may be an essential data acceptance factor.

The 3-year, Florida-specific use case offered a fresh viewpoint of using regulatory thresholds through a probabilistic lens using only state-held data (e.g., no NCCA data). While we could not complete the complex assessment methodology that FDEP uses to meet their reporting requirements, we could highlight the possible utility of the pseudo-probabilistic approach to strengthen state or local baseline ecological assessments when a formal, comprehensive estuarine monitoring program does not exist. The assessment similarities between the 2015–2017 and 2017–2019 assessment periods, specifically related to the physical characteristic parameters, i.e., SAL, PH, and TEMP, support our assumption that place- or issue-based placement of sampling sites potentially reduced data variability, which could mask evidence of ecological regime shifts and impact the effectiveness of estuarine resource management strategies (Carstensen et al., 2011; Elliott & Quintino, 2007; Gibbs, 2013). Conversely, the significant change in area “Not Sampled” for TN and TP and the uptick in area exceeding DO thresholds might reflect a shift in sampling strategy in the 2017–2019 reporting period. However, it must be noted that the study area was impacted by a category 5 hurricane in 2018 (“Hurricane Michael—October 2018”, https://www.weather.gov/mob/michael), which may have contributed to a greater proportion of the area being sampled during the following index period. Similarly, the change analysis afforded a statistically viable way to focus specifically on observed changes in water quality conditions over the two reporting periods. Information such as this could be used to monitor the trajectory of ecological change and indicate when new, more adaptive management strategies are needed to sustain or improve ecosystem resilience in estuaries (Pelletier et al., 2020).

Finally, the performance of the pseudo-probabilistic approach is promising. The CDF distribution means calculated from probabilistic estimates for each parameter closely aligned with actual mean values, suggesting that the sample population of the pseudo-probabilistic design generally approximated values observed in the raw data from 2015 and 2015–2019. For the 1-year and 5-year assessments, the pseudo-probabilistic error estimates were markedly better than the standard deviation of observed means. Generally, the results met the 90% confidence target, an assessment reliability benchmark used in NCCA reporting. The only exceptions were mean differences and error estimates produced for the 2015 and 2015–2019 ENTERO parameters, which may be attributed to sample collection locales (shoreline/upstream vs. open water). In this study, the similarities among actual versus estimated parameter means generally support the theory that probabilistic sampling produces the same results with less field effort (Carstensen, 2007; Olsen et al., 1997; Overton & Stehman, 1996; Stevens, 1994).

## Conclusion

Estuarine resource managers are continually challenged with balancing the need to address statute and regulatory monitoring and track system-wide trends in ecological condition. While estuaries are resilient to natural perturbations (e.g., freshwater inundation and hurricanes), climate change and anthropogenic stressors (e.g., land-use and contamination) increase the likelihood that the beneficial services that estuaries provide (e.g., storm surge mitigation, economic, and recreational) will be diminished—ultimately decreasing the resilience of both ecological and human ecosystems over the long term. As previously noted, many estuarine management programs lack the capacity to conduct statistically robust, system-wide ecological assessments. Such information is needed to recognize the patterns that may suggest that estuarine conditions are reaching an ecological tipping point from which the cost of recovery would be great, if achievable at all.

The pseudo-probabilistic approach offers a statistically plausible, readily transferable way to use secondary data to increase information about ecological conditions in estuaries without compromising existing monitoring priorities. By leveraging existing tools, this method allows resource managers to quickly produce estuarine condition assessments using data on hand or other sources in a manner consistent with EPA’s NCCA program—a highly successful national coastal monitoring program. Results produced by the pseudo-probabilistic design offer state and local programs complementary information about the extent of ecological conditions in estuarine systems. At a minimum, estimates showing the amount of area “Not Sampled” offer some indication that additional data may need to be collected in unsampled areas to improve the interpretation of assessment results or more accurately characterize the whole resource. While there is no substitute for statistically and spatially robust field studies, the pseudo-probabilistic approach may serve as an intermediate alternative to help fill critical knowledge gaps in ecological baseline conditions to support efforts in sustaining or improving the long-term resilience of our estuaries.

### Supplementary Information

Below is the link to the electronic supplementary material.

Supplementary file1 (ZIP 2689 kb)

## Data Availability

The authors declare that the data supporting the findings of this study are available within the paper and/or its supplementary information file.
