# Supplementary material for: Development of a generalized pseudo-probabilistic approach for characterizing ecological conditions in estuaries using secondary data
Source: Environ Monit Assess. 2024 Jul 20;196(8):753. doi: 10.1007/s10661-024-12877-8 (PMC11271375; doi:10.1007/s10661-024-12877-8)
Supplement: Supplementary file 1 — Supplementary file1 (ZIP 2689 kb) [file 10661_2024_12877_MOESM1_ESM.zip › mapData/NWFWMD_metadata.htm]

 

# Northwest FLorida Water Management District - Estuaries (Subset from the US EPA's Estuarine Resource Features Dataset)

Tags There are no tags for this item.

### Summary

```
This geospatial dataset provides location-specific information on estuarine features within the "Northwest Florida Water Management District (NWFWMD)" subset. This geospatial resource supports research associated with demonstrations presented in "Harwell, L. et al., Development of a generalized pseudo-probabilistic approach for characterizing ecological conditions in estuaries using secondary data."
```

### Description

```
This GIS feature layer or sample frame was created to represent the study area described in "Harwell, L. et al., Development of a generalized pseudo-probabilistic approach for characterizing ecological conditions in estuaries using secondary data" and to facilitate spatial analysis. The sample frame was extracted from the United States Geological Survey’s 1:100,000 digital line graph (DLG). Originally, the open water portion of the DLG was enhanced to support the US Environmental Protection Agency's (EPA) Environmental Monitoring and Assessment Program - Estuaries (1991-1994) and National Coastal Assessment (2000-2006). The resulting DLG enhancement was previously created to include all US marine tidally influenced waters and the adjacent nearshore open ocean (Bourgeois et al., 1998). Subsequent use of this dataset has been adapted to support EPA's National Coastal Condition Assessment (2010 - current). For this presentation of features, the nearshore polygons within the sample frame provided the seaward boundary. Salinity zone polygons (Nelson, 2015) were added to identify the upstream boundary of each estuarine system. Finally, a GIS shapefile of NWFWMD county boundaries (“Northwest Florida Water Management District Boundaries with county divisions," https://nwfwmd-open-data-nwfwmd.hub.arcgis.com/datasets/NWFWMD::nwfwmd-county-boundaries/about) was used to delineate the east-west extent of the study area. A straight line, extending from the shoreline to the outermost seaward polygon, was used to bisect a large open water feature (Apalachee Bay) managed by two different Florida water management districts. The sample frame for the study area encompassed a total estuarine area of 2705.90 km2. Please note: NOAA's Coastal Assessment Framework (CAF, 2015) coastal watershed drainage area attributes were added as supplementary informaiton. For more information about NOAA's CAF, please visit: https://www.ncei.noaa.gov/archive/archive-management-system/OAS/bin/prd/jquery/project/details/703.
```

### Credits

```
Bourgeois, P.E., V.J. Sclafani, J.K. Summers and S.C. Robb. 1998. Think before you sample data. GEOWorld, December, 1998: 4pp; Nelson, D. M. NOAA National Ocean Service (2015). Estuarine Salinity Zones in US East Coast, Gulf of Mexico, and US West Coast from 1999-01-01 to 1999-12-31 (NCEI Accession 0127396). NOAA National Centers for Environmental Information. Dataset. https://www.ncei.noaa.gov/archive/accession/0127396.
```

### Use limitations

```
Please check sources, scale, accuracy, currency and other available information, and confirm that you are using the most recent copy of both data and metadata.
```

### Extent

| West | -87.5 | East | -84.1 |
| North | 30.8 | South | 29.6 |

### Scale Range

| Maximum (zoomed in) | 1:5,000 |
| Minimum (zoomed out) | 1:150,000,000 |

### Topics and Keywords  ▼►

Themes or categories of the resource Boundaries, Inland Waters, Oceans
  

Content type ⇔ Downloadable Data
:   Export to FGDC CSDGM XML format as Resource Description No
  

Theme keywords 020:097
:   Thesaurus's language English (UNITED STATES)

    Thesaurus ▼►

    :   Title Federal Program Inventory

        Publication date 2013-09-16
          

        Resource location online
        :   Online location (URL) https://www.performance.gov/federalprograminventory

            Name Federal Program Inventory

            Function performed information

Theme keywords Estuary, Marine, Water
:   Thesaurus's language English (UNITED STATES)

    Thesaurus ▼►

    :   Title EPA GIS Keyword Thesaurus

        Publication date 2007-11-02
          

        Resource location online
        :   Online location (URL) https://www.epa.gov/geospatial/epa-metadata-technical-specification

            Name EPA Metadata Technical Specification

            Function performed information

Place keywords Florida
:   Thesaurus's language English (UNITED STATES)

    Thesaurus ▼►

    :   Title EPA Place Names

        Publication date 2015-01-31
          

        Resource location online
        :   Online location (URL) https://ofmpub.epa.gov/sor\_internet/registry/termreg/searchandretrieve/taxonomies/search.do?search=&searchString=&taxonomyName=WBT%20-%20Geographic%20Locations

            Name Web Taxonomy - Geographic Locations

            Function performed information

### Citation  ▼►

Title Combined Estuaries (NWFWMD Subset), United States and PR, USEPA/ORD/CEMM/GEMMD/EAB

Publication date 2022-09-06

### Citation Contacts  ▼►

Responsible party - point of contact
:   Individual's name Linda Harwell

    Organization's name USEPA/ORD/CEMM/GEMMD/EAB

    Contact's position IT Specialist
      
    :   Contact information ▼►

        :   Address
            :   Type

                e-mail address harwell.linda@epa.gov

### Resource Details  ▼►

Dataset languages ⇔ English (UNITED STATES)

Dataset character set utf8 - 8 bit UCS Transfer Format
  

Spatial representation type ⇔ vector
  

Supplemental information

:   ```
    Processing Environment for Resource & Metadata:
    Esri Inc. (2021). ArcGIS Pro (Version 2.9.0). Esri Inc. https://www.esri.com/en-us/arcgis/products/arcgis-pro/overview

    Processing Environment for Interactive R Script:
    R Core Team, 2021. R: A language and environment for statistical computing. R Foundation for Statistical Computing, Vienna, Austria. URL: https://cran.r-project.org/developed)
    R Studio Team, 2021. RStudio: Integrated Development for R. RStudio. PBC, Boston, MA. URL: https://www.rstudio.com/
    ```

Processing environment ⇔ Microsoft Windows 10 Version 10.0 (Build 19042) ; Esri ArcGIS 12.9.0.32739
  

Credits

:   ```
    Bourgeois, P.E., V.J. Sclafani, J.K. Summers and S.C. Robb. 1998. Think before you sample data. GEOWorld, December, 1998: 4pp.
    ```

ArcGIS item properties
:   Name ⇔ Combined\_Subsets\_20220830

    Size ⇔ 0.000

    Location ⇔ file://\\AA\ORD\GBR\Data\Priv\SHC\_264\Andrea\NCCASD\D2\_GIS\Combined\_Subsets\_20220830.shp
    :   Access protocol ⇔ Local Area Network

### Extents  ▼►

Extent
:   Description

    :   ```
        In terms of spatial extent, these estuarine features can be found within the "NWFWMD" - the bounding box encompasses this extent.

        As for temporal extent, it is unclear when data from each source was collected. National and regional base maps used to generate the total water-surface area for the nation’s estuarine resources (USGS EROS) were most likely created sometime in the late 1980's. As stated under "Lineage" within the "Data Source" section, this work aligned with NOAA’s Continually Updated Shoreline Product (NOAA CUSP) and was used by US EPA’s National Aquatic Resource Surveys (NARS), National Coastal Condition Assessment (NCCA). Given this, at some point in time, estuarine resource info from USGS EROS and data from NOAA's CUSP was extracted by NARS and manipulated to some degree. No time frame was recorded during the creation of this "new" spatial data product so it would not be appropriate to provide one here. For more information, please visit the links listed within the "Data Source" section and/or contact Linda Harwell (main point of contact) via email at harwell.linda@epa.gov.
        ```

    Geographic extent
    :   Bounding rectangle
        :   Extent type Extent used for searching

            West longitude -87.5

            East longitude -84.1

            South latitude 29.6

            North latitude 30.8

            Extent contains the resource Yes

### Resource Points of Contact  ▼►

Point of contact - publisher
:   Individual's name Linda Harwell

    Organization's name USEPA/ORD/CEMM/GEMMD/EAB

    Contact's position IT Specialist
      
    :   Contact information ▼►

        :   Address
            :   Type

                e-mail address harwell.linda@epa.gov

Point of contact - processor
:   Individual's name Andrea Lamper

    Organization's name USEPA/ORD/CEMM/GEMMD/EAB

    Contact's position ArcGIS Data Specialist (ORAU SSC)
      
    :   Contact information ▼►

        :   Address
            :   Type

                e-mail address lamper.andrea@epa.gov

### Resource Maintenance  ▼►

Resource maintenance
:   Update frequency as needed
      

    Maintenance contact - point of contact
    :   Individual's name Linda Harwell

        Organization's name USEPA/ORD/CEMM/GEMMD/EAB

        Contact's position IT Specialist
          
        :   Contact information ▼►

            :   Address
                :   Type

                    e-mail address harwell.linda@epa.gov

### Resource Constraints  ▼►

Constraints
:   Limitations of use

    :   ```
        Please check sources, scale, accuracy, currency and other available information, and confirm that you are using the most recent copy of both data and metadata.
        ```

Security constraints
:   Classification unclassified

Legal constraints
:   Limitations of use

    :   ```
        EPA Public Domain License
        ```

    Access constraints unrestricted license
      

    Other constraints

    :   ```
        https://edg.epa.gov/EPA_Data_License.html
        ```

### Spatial Reference  ▼►

ArcGIS coordinate system
:   Type ⇔ Projected

    Geographic coordinate reference ⇔ GCS\_North\_American\_1983

    Projection ⇔ Albers

    Coordinate reference details ⇔
    :   ProjectedCoordinateSystem
        :   XOrigin -16901100

            YOrigin -8579000

            XYScale 266467840.99085236

            ZOrigin -100000

            ZScale 10000

            MOrigin -100000

            MScale 10000

            XYTolerance 0.001

            ZTolerance 0.001

            MTolerance 0.001

            HighPrecision true

            WKT PROJCS["Albers",GEOGCS["GCS\_North\_American\_1983",DATUM["D\_North\_American\_1983",SPHEROID["GRS\_1980",6378137.0,298.257222101]],PRIMEM["Greenwich",0.0],UNIT["Degree",0.0174532925199433]],PROJECTION["Albers"],PARAMETER["false\_easting",0.0],PARAMETER["false\_northing",0.0],PARAMETER["central\_meridian",-96.0],PARAMETER["standard\_parallel\_1",29.5],PARAMETER["standard\_parallel\_2",45.5],PARAMETER["latitude\_of\_origin",37.5],UNIT["Meter",1.0]]
  

Reference system identifier
:   Value 4269

### Spatial Data Properties  ▼►

Vector ▼►

:   Level of topology for this dataset ⇔ geometry only
      

    Geometric objects
    :   Feature class name Combined\_Subsets\_20220830

        Object type ⇔ composite

        Object count ⇔ 0

ArcGIS Feature Class Properties ▼►

:   Feature class name Combined\_Subsets\_20220830
    :   Feature type ⇔ Simple

        Geometry type ⇔ Polygon

        Has topology ⇔ FALSE

        Feature count ⇔ 0

        Spatial index ⇔ FALSE

        Linear referencing ⇔ FALSE

### Data Quality  ▼►

Scope of quality information ▼►

:   Resource level dataset

Data quality report - Completeness commission  ▼►

:   Conformance test results
    :   Test passed Yes

        Result explanation

        :   ```
            The completeness of the data reflects the content of the sources and may not include a complete estuarine dataset.
            ```

        Product specification ▼►

        :   Title N/A
              

            Responsible party - point of contact
            :   Individual's name Linda Harwell

                Organization's name USEPA/ORD/CEMM/GEMMD/EAB

                Contact's position IT Specialist
                  
                :   Contact information ▼►

                    :   Address
                        :   Type

                            e-mail address harwell.linda@epa.gov

### Lineage  ▼►

Lineage statement

:   ```
    *Note: The below statement and following process steps, excluding the last process step, relate to the creation of the combined or master estuaries dataset. See last process step for information on creation of NWFWMD dataset.*

    A majority of the following process steps relate to actions completed prior to appending one dataset to another. Such actions include: limiting existing data, creating subsets, and renaming fields. The goal was to create and offer one master estuarine dataset, as well as geo-region subsets of this master, for use within the project related interactive script.
    ```

Process step ▼►

:   When the process occurred 2020-08-27 00:00:00

    Description

    :   ```
        A definition query to omit all "Land" values from the "ESTUARIES" field was applied to the original shapefile called "us_estuaries" (57,702 features total). This query brought the total number of estuarine features down to 4,193. 

        Once queried, this original shapefile was exported as a new feature class called "us_estuaries_20200827".
        ```

    Rationale

    :   ```
        "Land" values were omitted because only water features were of interest.

        An export was needed to ensure that the definition query would be "permanent" and that a new subset would be created.
        ```

    Process contact - processor
    :   Individual's name Andrea Lamper

        Organization's name USEPA/ORD/CEMM/GEMMD/EAB

        Contact's position ArcGIS Data Specialist (ORAU SSC)
          
        :   Contact information ▼►

            :   Address
                :   Type

                    e-mail address lamper.andrea@epa.gov

Process step ▼►

:   When the process occurred 2020-08-27 00:00:00

    Description

    :   ```
        A number of fields were renamed within this newly created feature class ("us_estuaries_20200827") - see below for changes. Note, if a field is not listed here, then no changes were made.

        ESTUARIES to SYSTEM_NM
        STATE_NAME to PSTL_CODE
        SQ_KILOMET to AREA_KM2
        REGION to GEO_REGION
        ```

    Rationale

    :   ```
        Field names were revised to match target feature class for ease of use when running the append tool - covered in following process steps.
        ```

    Process contact - processor
    :   Individual's name Andrea Lamper

        Organization's name USEPA/ORD/CEMM/GEMMD/EAB

        Contact's position ArcGIS Data Specialist (ORAU SSC)
          
        :   Contact information ▼►

            :   Address
                :   Type

                    e-mail address lamper.andrea@epa.gov

Process step ▼►

:   When the process occurred 2020-08-28 00:00:00

    Description

    :   ```
        After fields were renamed, "us_estuaries_20200827" was limited to just those estuarine features in AK, HI, and PR via a definition query. 

        With 2,198 features remaining, this feature class was exported and named "us_estuaries_20200828".
        ```

    Rationale

    :   ```
        The target feature class, or the one "us_estuaries_20200828" is being appended to, covered the continental US but lacked data for AK, HI, and PR, so data from this feature class filled these gaps. 

        An export was needed to ensure that the definition query would be "permanent" and that a new subset would be created.
        ```

    Process contact - processor
    :   Individual's name Andrea Lamper

        Organization's name USEPA/ORD/CEMM/GEMMD/EAB

        Contact's position ArcGIS Data Specialist (ORAU SSC)
          
        :   Contact information ▼►

            :   Address
                :   Type

                    e-mail address lamper.andrea@epa.gov

Process step ▼►

:   When the process occurred 2020-08-28 00:00:00

    Description

    :   ```
        The other original feature class called "NCCA_2020_21Coastal_20180917" was exported and called "NCCA_2020_21Coastal_Copy20200828". 

        Fields within "NCCA_2020_21Coastal_Copy20200828" were renamed to match those within the input feature class - see below for changes.

        WTBDY_NM to SYSTEM_NM
        AREA_SQKM to AREA_KM2
        NCCA_REG to GEO_REGION

        *The renamed field listed below did not relate to harmonizing the two datasets, since it only existed in the NCCA feature class. Note, if a field is not listed here, then no changes were made.

        EDADR_CODE to EDADRA
        ```

    Rationale

    :   ```
        "NCCA_2020_21Coastal_20180917" was exported to create a copy of the original data.

        Fields were renamed in "NCCA_2020_21Coastal_Copy20200828" and "us_estuaries_20200827" to match one another. Reflecting on this step, please note that field mapping can be done within the append tool so that fields with different names can be matched and stitched together. Renaming of fields can be done afterwards.
        ```

    Process contact - processor
    :   Individual's name Andrea Lamper

        Organization's name USEPA/ORD/CEMM/GEMMD/EAB

        Contact's position ArcGIS Data Specialist (ORAU SSC)
          
        :   Contact information ▼►

            :   Address
                :   Type

                    e-mail address lamper.andrea@epa.gov

Process step ▼►

:   When the process occurred 2020-08-28 00:00:00

    Description

    :   ```
        The append tool was run with the input feature class being "us_estuaries_20200828" and the target feature class being "NCCA_2020_21Coastal_Copy20200828".

        Note, the append tool does not create an output since data is stitched to an existing dataset, thus "NCCA_2020_21Coastal_Copy20200828" was exported and called "Combined_Estuaries_20200828".
        ```

    Rationale

    :   ```
        The append tool was run to bring these two datasets together. After which, it needed to be exported to create the master dataset.
        ```

    Process contact - processor
    :   Individual's name Andrea Lamper

        Organization's name USEPA/ORD/CEMM/GEMMD/EAB

        Contact's position ArcGIS Data Specialist (ORAU SSC)
          
        :   Contact information ▼►

            :   Address
                :   Type

                    e-mail address lamper.andrea@epa.gov

Process step ▼►

:   When the process occurred 2020-08-28 00:00:00

    Description

    :   ```
        Geo-region subsets were created from this "Combined_Estuaries_20200828" feature class by querying for each individual region value within the "GEO_REGION" field. With each query, the limited feature class was exported as a shapefile and named after the region it represented.

        Unnecessary fields within these geo-region subsets were omitted upon export - see below for deleted fields. Note, if a field is not listed here, then it was not omitted.

        CAF_ID
        CAF_REGION
        EDACDA
        EDACDA_NM
        EDASUBEDA
        SUBEDA_NM
        EDADRA
        NMN_EDACDA
        NMN_SUBEDA
        ```

    Rationale

    :   ```
        Geo-region subsets were created for ease of use within the project related interactive script. The idea was that users may only want to look at one specific region and/or may also not have the bandwidth to run large datasets through the tool. Additionally, a number of fields were deemed irrelevant so they were omitted from final geo-region subsets.
        ```

    Process contact - processor
    :   Individual's name Andrea Lamper

        Organization's name USEPA/ORD/CEMM/GEMMD/EAB

        Contact's position ArcGIS Data Specialist (ORAU SSC)
          
        :   Contact information ▼►

            :   Address
                :   Type

                    e-mail address lamper.andrea@epa.gov

Process step ▼►

:   When the process occurred 2021-07-28 00:00:00

    Description

    :   ```
        A copy of a shapefile called "NWFWMD_County_Boundaries" (provided to me by Linda Harwell) was created. Also, a copy of the shapefile "Combined_Estuaries_20200828" was created as well. This estuaries copy was edited - essentially, two features' vertices were edited to "cut" the shapefile right at the NWFWMD county boundary copy's most eastern edge. After these minor edits were made, three select by location trials were run to omit unwanted estuaries from the final shapefile. This final shapefile depicting only estuaries in the NWFWMD is called "NWFWMD_Estuaries_20210728".
        ```

    Rationale

    :   ```
        This process was run because a subset of NWFWMD estuaries was needed & requested by Linda Harwell.
        ```

    Process contact - processor
    :   Individual's name Andrea Lamper

        Organization's name USEPA/ORD/CEMM/GEMMD/EAB

        Contact's position ArcGIS Data Specialist (ORAU SSC)
          
        :   Contact information ▼►

            :   Address
                :   Type

                    e-mail address lamper.andrea@epa.gov

Process step ▼►

:   When the process occurred 2022-07-14 00:00:00

    Description

    :   ```
        An unnecessary field called "MergeID" was recognized within these geo-region subsets. As requested, this field was deleted by running the batch delete field tool on each subset. After which, the batch feature class to shapefile tool was run to export all revised subsets.
        ```

    Rationale

    :   ```
        Irrelevant field needed to be deleted from subsets.
        ```

    Process contact - processor
    :   Individual's name Andrea Lamper

        Organization's name USEPA/ORD/CEMM/GEMMD/EAB

        Contact's position ArcGIS Data Specialist (ORAU SSC)
          
        :   Contact information ▼►

            :   Address
                :   Type

                    e-mail address lamper.andrea@epa.gov

Process step ▼►

:   When the process occurred 2022-08-30 00:00:00

    Description

    :   ```
        Revised geo-region subsets were merged and called "Combined_Subsets_20220830" to create a final master dataset.
        ```

    Rationale

    :   ```
        This step was completed for use within project related interactive script.
        ```

    Process contact - processor
    :   Individual's name Andrea Lamper

        Organization's name USEPA/ORD/CEMM/GEMMD/EAB

        Contact's position ArcGIS Data Specialist (ORAU SSC)
          
        :   Contact information ▼►

            :   Address
                :   Type

                    e-mail address lamper.andrea@epa.gov

Source data ▼►

:   Description

    :   ```
        National and regional base maps were derived using 1:100,000 digital line graph hydrology quadrangles (USGS EROS) to generate the total water-surface area for the nation’s estuarine resources and aligned with NOAA’s CUSP used by US EPA’s NARS, NCCA (https://www.epa.gov/national-aquatic-resource-surveys). See below for a link to each source.
        ```

    Source citation ▼►

    :   Title USGS EROS. Digital Line Graphs (DLGs) - Intermediate Scale
          

        Resource location online
        :   Online location (URL) https://www.usgs.gov/centers/eros/science/usgs-eros-archive-digital-line-graphs-dlgs-intermediate-scale

            Description Digital Object Identifier (DOI) number: /10.5066/F7RN369N

    Source citation ▼►

    :   Title NOAA CUSP. Continually Updated Shoreline Product
          

        Resource location online
        :   Online location (URL) https://shoreline.noaa.gov/data/datasheets/cusp.html

### Fields  ▼►

Details for object Combined\_Subsets\_20220830 ▼►

:   Type ⇔ Feature Class

    Row count ⇔ 0

    Definition

    :   ```
        Estuarine dataset for 27 coastal states within the US states and PR
        ```

    Definition source

    :   ```
        Point of Contact
        ```

    Field FID ▼►

    :   Alias ⇔ FID

        Data type ⇔ OID

        Width ⇔ 4

        Precision ⇔ 0

        Scale ⇔ 0
          

        Field description ⇔

        :   ```
            Internal feature number.
            ```

        Description source ⇔

        :   ```
            Esri
            ```

        Description of values ⇔

        :   ```
            Sequential unique whole numbers that are automatically generated.
            ```

    Field Shape ▼►

    :   Alias ⇔ Shape

        Data type ⇔ Geometry

        Width ⇔ 0

        Precision ⇔ 0

        Scale ⇔ 0
          

        Field description ⇔

        :   ```
            Feature geometry.
            ```

        Description source ⇔

        :   ```
            Esri
            ```

        Description of values ⇔

        :   ```
            Coordinates defining the features.
            ```

    Field PSTL\_CODE ▼►

    :   Alias ⇔ PSTL\_CODE

        Data type ⇔ String

        Width ⇔ 80

        Precision ⇔ 0

        Scale ⇔ 0
          

        Field description

        :   ```
            Postal code or state abbreviation.
            ```

        Description source

        :   ```
            Point of Contact
            ```

        Description of values

        :   ```
            There are 25 unique values listed within this field.
            Examples: "SC", "PR"
            ```

    Field GEO\_REGION ▼►

    :   Alias ⇔ GEO\_REGION

        Data type ⇔ String

        Width ⇔ 80

        Precision ⇔ 0

        Scale ⇔ 0
          

        Field description

        :   ```
            Geographic region for which estuary is located in.
            ```

        Description source

        :   ```
            Point of Contact
            ```

        Description of values

        :   ```
            There are 10 unique values listed within this field.
            Examples: "Southeast", "Beaufort Sea"
            ```

    Field PROVINCE ▼►

    :   Alias ⇔ PROVINCE

        Data type ⇔ String

        Width ⇔ 80

        Precision ⇔ 0

        Scale ⇔ 0
          

        Field description

        :   ```
            Administrative division boundary.
            ```

        Description source

        :   ```
            Point of Contact
            ```

        Description of values

        :   ```
            There are 12 unique values listed within this field.
            Examples: "Carolinian Province", "Arctic"
            ```

    Field SYSTEM\_NM ▼►

    :   Alias ⇔ SYSTEM\_NM

        Data type ⇔ String

        Width ⇔ 80

        Precision ⇔ 0

        Scale ⇔ 0
          

        Field description

        :   ```
            Estuarine system name.
            ```

        Description source

        :   ```
            Point of Contact
            ```

        Description of values

        :   ```
            There are 2,351 unique values listed within this field.
            Examples: "Dry Bay", "Little River"
            ```

    Field FEAT\_NM ▼►

    :   Alias ⇔ FEAT\_NM

        Data type ⇔ String

        Width ⇔ 80

        Precision ⇔ 0

        Scale ⇔ 0
          

        Field description

        :   ```
            Estuarine feature name.
            ```

        Description source

        :   ```
            Point of Contact
            ```

        Description of values

        :   ```
            There are 1,713 unique values listed within this field.
            Examples: "Bass Harbor", "Great Bay"
            ```

    Field AREA\_KM2 ▼►

    :   Alias ⇔ AREA\_KM2

        Data type ⇔ Double

        Width ⇔ 19

        Precision ⇔ 0

        Scale ⇔ 0
          

        Field description

        :   ```
            Area in square kilometers.
            ```

        Description source

        :   ```
            Point of Contact
            ```

        Description of values

        :   ```
            Unit of measurement.
            ```

    Field SALINITY\_Z ▼►

    :   Alias ⇔ SALINITY\_Z

        Data type ⇔ String

        Width ⇔ 80

        Precision ⇔ 0

        Scale ⇔ 0
          

        Field description

        :   ```
            Estuarine salinity zone.
            ```

        Description source

        :   ```
            Point of Contact
            ```

        Description of values

        :   ```
            There are 5 unique values listed within this field.
            Examples: "Coastal Zone", "Tidal Fresh Zone"
            ```

    Field Shape\_Leng ▼►

    :   Alias ⇔ Shape\_Leng

        Data type ⇔ Double

        Width ⇔ 19

        Precision ⇔ 0

        Scale ⇔ 0
          

        Field description

        :   ```
            Length of feature in internal units.
            ```

        Description source

        :   ```
            Esri
            ```

        Description of values

        :   ```
            Lengths that are automatically generated.
            ```

    Field Shape\_Area ▼►

    :   Alias ⇔ Shape\_Area

        Data type ⇔ Double

        Width ⇔ 19

        Precision ⇔ 0

        Scale ⇔ 0
          

        Field description ⇔

        :   ```
            Area of feature in internal units squared.
            ```

        Description source ⇔

        :   ```
            Esri
            ```

        Description of values ⇔

        :   ```
            Positive real numbers that are automatically generated.
            ```

### Metadata Details  ▼►

Metadata language ⇔ English (UNITED STATES)

Metadata character set utf8 - 8 bit UCS Transfer Format
  

Metadata identifier 89D66D2A-DD88-47AA-A891-6B4A88301C5D
  

Scope of the data described by the metadata ⇔ dataset

Scope name ⇔ dataset
  

Last update 2022-09-07
  

ArcGIS metadata properties
:   Metadata format ArcGIS 1.0

    Standard or profile used to edit metadata ISO19115\_3
      

    Created in ArcGIS for the item 2018-10-02 15:43:26

    Last modified in ArcGIS for the item 2022-09-07 12:02:39
      

    Automatic updates
    :   Have been performed Yes

        Last update 2022-08-30 09:22:41

### Metadata Contacts  ▼►

Metadata contact - point of contact
:   Individual's name Linda Harwell

    Organization's name US EPA, Office of Research and Development, Center for Environmental Measurement and Modeling, Gulf Ecosystem Measurement and Modeling Division, Ecosystem Assessment Branch

    Contact's position IT Specialist
      
    :   Contact information ▼►

        :   Address 1 Sabine Island Dr, Gulf Breeze, FL 32561
            :   Type

                e-mail address harwell.linda@epa.gov

### Metadata Maintenance  ▼►

Maintenance
:   Update frequency as needed
      

    Maintenance contact - point of contact
    :   Individual's name Linda Harwell

        Organization's name US EPA, Office of Research and Development, Center for Environmental Measurement and Modeling, Gulf Ecosystem Measurement and Modeling Division, Ecosystem Assessment Branch

        Contact's position Research Principle Investigator
          
        :   Contact information ▼►

            :   Address
                :   Type

                    e-mail address harwell.linda@epa.gov

### Thumbnail and Enclosures  ▼►

Thumbnail
:   Thumbnail type
