# Supplementary material for: Development of a generalized pseudo-probabilistic approach for characterizing ecological conditions in estuaries using secondary data
Source: Environ Monit Assess. 2024 Jul 20;196(8):753. doi: 10.1007/s10661-024-12877-8 (PMC11271375; doi:10.1007/s10661-024-12877-8)
Supplement: Supplementary file 1 — Supplementary file1 (ZIP 2689 kb) [file 10661_2024_12877_MOESM1_ESM.zip › Draft_HarwellL_etal_PseudoProbAssess_Suppl.html]

Development of a generalized pseudo-probabilistic approach for characterizing ecological conditions in estuaries using secondary data.


# Development of a generalized pseudo-probabilistic approach for characterizing ecological conditions in estuaries using secondary data.

#### Harwell, L., McMillion, C., Lamper, A., & Summers, J.K

#### 11 June, 2024

Corresponding Author: Linda Harwell Affiliation: Gulf Ecosystem
Measurement and Modeling Division, 1 Sabine Island Drive, Gulf Breeze,
FL 32561 Email: harwell.linda@epa.gov

# Contents

Supplementary publication material including raw code snippets.

## Creating the Pseudo-Probabilistic Framework

The following raw code shows the steps used to create the hexagon
grid for summarizing secondary data. It also offers the steps for
merging the GRTS generated survey design sites with the hexagon grid to
produce the pseudo-probabilistic framework.

```
## How it was done

# Determine size and diameter of hexagon cells
totarea = as.numeric(st_area(sframe))
totarea_km2 = totarea/1000000
n_hex <- 45 # Number of desired sites x 1.5 to account the random select process.
tmp_area <- totarea/n_hex
cell_area <- as.integer(2 * sqrt(( 2 * tmp_area) / (3 * sqrt(3))))

# Minimize spatial buffer around sampleframe polygon
resourceBuffer <- st_buffer(sframe, dist=0)

# For reproducibility
set.seed(258369)

# Produce grid overlay
hex_overlay <- st_make_grid(resourceBuffer, cellsize = cell_area, what = "polygons", square = FALSE) %>%
 st_as_sf() %>%
 mutate(area = st_area(.))

hex_overlay$centroid <- st_centroid(hex_overlay$x) %>% st_transform(., st_crs(sframe))
st_geometry(hex_overlay) <- "geometry"

## Extract centroid Lat/Longs from overlay
hex_overlay$x <- st_geometry(hex_overlay$centroid) %>% st_transform(., '+proj=longlat +datum=WGS84')

hex_overlay <- hex_overlay %>%
  mutate(clong = unlist(map(hex_overlay$x,1)),
         clat = unlist(map(hex_overlay$x,2)))

# Clip overlay to sample frame
rsrc_grid <- st_intersection(hex_overlay, sframe) %>%
    st_as_sf() %>%
    mutate(area = st_area(.),
           n_reshex = n())

rsrc_grid$hex_id <- seq.int(nrow(rsrc_grid))
rsrc_grid$area <- as.numeric(rsrc_grid$area)

# GRTS

set.seed(258369)
prob <- grts(sframe, n_base = n_hex)

extract_base <- prob$sites_base

## Pseudo-prob design
# Only distinct resource grid hexagons are keep to account for differences
# between the GRTS site selection process and the number of 
# hexagon grid cells available.

design_in <- st_join(extract_base, rsrc_grid, join=st_within) %>%
  distinct(hex_id, .keep_all=TRUE) %>%
  rename(dsgn_site=siteID) %>%
  select(-c(centroid, x))

design <- design_in %>% st_drop_geometry()
```

## Mann-Whitney-U/Wilcoxan Tests (MWU): 2015 FWIN - NCCA

Supporting Information for: Table 5

Wilcoxon rank sum with continuity correction or Mann-Whitney U (MWU)
analyses were performed to identify to differences in the distribution
of parameter values between the two sources of secondary data.

Featured function(s):

- ‘ggbetweenstats’ {ggstatsplot}

Reference:

Patil, I. (2021). Visualizations with statistical details: The
‘ggstatsplot’ approach. Journal of Open Source. Software, 6(61), 3167,
doi:10.21105/joss.03167

```
## How it was done

for (i in cdf_vars) {

n1 <- which(cdf_vars == i)

# Prepare data
mwu_2015 <- points_df %>%
  filter(parameter == i & actyear == 2015 & !is.na(result)) %>%
  mutate(survey_type = ifelse(survey_type == "FWIN", "FWIN 2015", "NCCA 2015")) %>%
  group_by(as.factor(survey_type))

# Mann-Whitney-Wilcoxan/U 2015 - Used to verify stats produced by 'ggstatsplot'
# print(i)
# print(mwu_2015 <- wilcox.test(result ~ survey_type, data=mwu_2015, exact=FALSE))

# Mann-Whitney via 'ggstatsplot'.
mplot <- ggbetweenstats( # independent samples)
  data = mwu_2015,
  x = survey_type,
  y = result,
  xlab="Data Source",
  ylab=paste0(plabels[n1]," (",punits[n1],")"),
  plot.type = "box", # for boxplot
  violin.args = list(width = 0), # to remove violin plot
  type = "nonparametric", # for wilcoxon
  centrality.plotting = TRUE # keep median reporting
)

# Save output using unique df names for plots
assign(paste0('mwu_',i), mplot)
}
```

### Chlorophyll a

### Diss. Oxygen

### Enterococci

### pH

### Salinity

### Temp

### Total N

### Total P

## Mann-Whitney-U/Wilcoxan Tests (MWU): 2015-2019 FWIN - NCCA

Supporting Information for: Table 5

We performed the same Mann-Whitney tests on all data collected during
the index period 2015-2019 to look for significant differences in
population means between the FWIN data and NCCA data even though NCCA
only offer data for one sampling year (2015).

This analysis was performed using the same procedure described in the
previous section of this supplmental, adjusted to reflect the full 5
year suite of data rather than only 2015. Box plot and Mann-Whitney
results are presented.

Reference:

Patil, I. (2021). Visualizations with statistical details: The
‘ggstatsplot’ approach. Journal of Open Source. Software, 6(61), 3167,
doi:10.21105/joss.03167

### Chlorophyll a

### Diss. Oxygen

### Enterococci

### pH

### Salinity

### Temp

### Total N

### Total P

## Aggregate Data for Demos

Supporting Information for: Figures 2 and 3

Secondary data were subset using the hexagon grid overly fitted to
the study area polygon. Data that fell within a cell of the overlay are
summarized (geometric mean) by parameter and sampling year. Parameter
values were previously compared to 25-50-75th percentile threshold
values and Florida water quality standards based threshold values. Data
were assigned a categorical labels of “Low”, “Moderate” or “High” and
“At/Below” or “Exceeds”, respectively. Sites, selected as part of the
pseudo-probability inclusion process, missing parameter data were
labeled “Not Sampled”.

The summarizing process compensated for data collected under
different sampling regimes and at different sampling frequencies. The
mean lat/long of all data included within a hexagon cell represented the
relative location where data were collected.

Below is the raw code used to produce the data aggregates that
informed condition assessment demonstrations. In the example provided,
two sets of data are created: one to represent available 2015 data for a
single year assessment and another housing all data collected during the
index period 2015-2019 for multi-year assessments.

```
## How it was done

## Create FWIN Only Aggregate

agg_fl <- points_df %>%
  filter(survey_type == "FWIN") %>%
  st_drop_geometry()
  
sum_fl <- agg_fl %>%
    group_by(survey_type, hex_id, parameter, actyear) %>%
    dplyr::summarize(rsltmean = exp(mean(log(result)))) %>%
    ungroup()

m_coords_fl <- agg_fl  %>%
  group_by(survey_type, hex_id, actyear) %>%
  dplyr::summarize(mean_lat = mean(dlat),
                   mean_lon = mean(dlon),
                   num_sites = n()) %>%
  mutate(lat = mean_lat,
         lon = mean_lon) %>%
  ungroup()

mean_fl_coords <- m_coords_fl %>%
  st_as_sf(coords = c("mean_lon", "mean_lat"), crs=4326) %>%
  st_transform(crs = st_crs(sframe))

assess_fl <- left_join(sum_fl, mean_fl_coords, by=c("survey_type", "hex_id",
                                                    "actyear")) %>%
  rename(result = rsltmean) %>%
  left_join(cutpoints) %>%
  mutate(cond = ifelse(is.na(result), "Not Sampled",
                       ifelse(result < q25, "Low",
                              ifelse(result >= q25 & result <= q75, "Moderate", "High"))),
         fl_cond = ifelse(is.na(result), "Not Sampled",
                          ifelse(result <= threshold, "At/Below", "Above"))) %>%
  rename(dlat = lat,
         dlon = lon) %>%
  filter(parameter != "NA") %>%
  select(-geometry)

assess_dat_fl <- left_join(assess_fl, design, by="hex_id") %>%
  mutate(survey_type = "FWIN",
         siteID = ifelse(is.na(actyear), paste0("Site-", hex_id), paste0(survey_type, actyear, "-", hex_id)))

## Create Combined Data Source Aggregate

agg_all <- points_df %>%
  st_drop_geometry()

sum_all <- agg_all %>%
    #group_by(survey_type, hex_id, parameter, actyear) %>%
    group_by(hex_id, parameter, actyear) %>%
    dplyr::summarize(rsltmean = exp(mean(log(result)))) %>%
    ungroup()

m_coords_all <- agg_all  %>%
  group_by(hex_id, actyear) %>%
  dplyr::summarize(mean_lat = mean(dlat),
                   mean_lon = mean(dlon),
                   num_sites = n()) %>%
  mutate(lat = mean_lat,
         lon = mean_lon) %>%
  ungroup()

mean_all_coords <- m_coords_all %>%
  st_as_sf(coords = c("mean_lon", "mean_lat"), crs=4326) %>%
  st_transform(crs = st_crs(sframe))

assess_all <- left_join(sum_all, mean_all_coords, by=c("hex_id",
                                                    "actyear")) %>%
  rename(result = rsltmean) %>%
  left_join(cutpoints) %>%
  mutate(cond = ifelse(is.na(result), "Not Sampled",
                       ifelse(result < q25, "Low",
                              ifelse(result >= q25 & result <= q75, "Moderate", "High"))),
         fl_cond = ifelse(is.na(result), "Not Sampled",
                          ifelse(result <= threshold, "At/Below", "Above"))) %>%
  rename(dlat = lat,
         dlon = lon) %>%
  filter(parameter != "NA") %>%
  select(-geometry)

assess_dat_all <- left_join(assess_all, design, by="hex_id") %>%
  mutate(survey_type = "Combined",
         siteID = ifelse(is.na(actyear), paste0("Site-", hex_id), paste0(survey_type, actyear, "-", hex_id)))

## Combine for Regular Use-Cases

assess_data <- bind_rows(assess_dat_fl, assess_dat_all)

## Create Florida-Specific Use-Case

agg_iwqr <- points_df %>%
  filter(survey_type == "FWIN") %>%
  mutate(orig_year = actyear,
         actyear = ifelse(actmon %in% c(6,7) & actyear == 2017, 2017,
                           ifelse(actmon %in% c(8,9) & actyear == 2017, 2019, actyear))) %>%
  st_drop_geometry()
  
sum_iwqr <- agg_iwqr %>%
    group_by(survey_type, hex_id, parameter, actyear) %>%
    dplyr::summarize(rsltmean = exp(mean(log(result)))) %>%
    ungroup()

m_coords_iwqr <- agg_iwqr  %>%
  group_by(survey_type, hex_id, actyear) %>%
  dplyr::summarize(mean_lat = mean(dlat),
                   mean_lon = mean(dlon),
                   num_sites = n()) %>%
  mutate(lat = mean_lat,
         lon = mean_lon) %>%
  ungroup()

mean_iwqr_coords <- m_coords_iwqr %>%
  st_as_sf(coords = c("mean_lon", "mean_lat"), crs=4326) %>%
  st_transform(crs = st_crs(sframe))

assess_iwqr <- left_join(sum_iwqr, mean_iwqr_coords, by=c("survey_type", "hex_id",
                                                    "actyear")) %>%
  rename(result = rsltmean) %>%
  left_join(cutpoints) %>%
  mutate(cond = ifelse(is.na(result), "Not Sampled",
                       ifelse(result < q25, "Low",
                              ifelse(result >= q25 & result <= q75, "Moderate", "High"))),
         fl_cond = ifelse(is.na(result), "Not Sampled",
                          ifelse(result <= threshold, "At/Below", "Above"))) %>%
  rename(dlat = lat,
         dlon = lon) %>%
  filter(parameter != "NA") %>%
  select(-geometry)

assess_dat_iwqr <- left_join(assess_iwqr, design, by="hex_id") %>%
  mutate(survey_type = "IWQR",
         siteID = ifelse(is.na(actyear), paste0("Site-", hex_id), paste0(survey_type, actyear, "-", hex_id)))
```

## Assessment Demonstrations

Resource wide water quality conditions assessments were produced.
Only parameters common to both data sources were used in these
assessments. There are four demonstrations presented in the article.

The same pseudo-probabilistic survey design framework is applied to
each suite of data to produce probability estimates. The inclusion
probability and weighting factor are equal. Four demonstration of the
approach in action are presented. The spatial balance of the
pseudo-probabilistically derived survey design was reviewed.

Featured function(s):

- ‘cat\_analysis’ {spsurvey}
- ‘cont\_analysis’ {spsurvey}
- ‘sp\_balance’ {spsurvey}
- ‘change\_analysis’ {spsurvey}

Referene(s):

Dumelle, Michael., Kincaid, T. M., Olsen, A. R., and Weber, M. H.
(2022). spsurvey: Spatial Sampling Design and Analysis. R package
version 5.3.0. Retrieved 17 November 2022, from https://cran.r-project.org/web/packages/spsurvey/index.html.

### Categorical Condition Assessment

Supporting Information for: Figures 5 - 7

Below is the raw code used to produce the condition assessment
results. The example provided generates the single-year (2015) results
generated to simulate a probability based assessment using the pseudo-
probabilistic approach. Other condition assessment analyses were
performed using the same code, modified only to select the appropriate
data groupings.

```
## 2015 Assessment
survey_loop <- c("FWIN", "Combined")

for (i in survey_loop) {

assess_clean <- assess_data %>%
  filter(actyear == 2015 & survey_type == i) %>% 
  select(c(survey_type, siteID, hex_id, parameter, actyear, result, dlat, dlon, cond))
  
extract_dat <- assess_clean %>%
  full_join(design, by="hex_id") %>%
  mutate(siteID = ifelse(is.na(siteID), dsgn_site, siteID),
         xlat=ifelse(is.na(dlat), lat_WGS84, dlat),
         xlon=ifelse(is.na(dlon), lon_WGS84, dlon)) %>%
  pivot_wider(id_cols = c("siteID", "hex_id", "survey_type", "siteuse", "actyear", "dlat", "dlon", "xlat", "xlon"),
              names_from = "parameter", values_from = c("result", "cond")) %>%
  mutate(across(where(is.character), ~replace_na(.x, "Not Sampled"))) %>%
  st_as_sf(coords = c("xlon", "xlat"), crs=4326) %>%
  st_transform(crs = st_crs(sframe)) %>%
  filter(siteuse == "Base") %>%
  select(-cond_NA) %>%
  distinct(c(siteID), .keep_all = TRUE)

data_2015 <- extract_dat  %>%
  mutate(sites_n = n_distinct(siteID),
         wgt=totarea_km2/sites_n)
  
# Examine the spatial balance of the design
sbal <- sp_balance(data_2015, sframe)
print(sbal)

est_out <- cat_analysis(
  data_2015,
  siteID = "siteID",
  vars = cat_vars,
  weight = "wgt"
  )

cdf_out <- cont_analysis(
  data_2015,
  siteID = "siteID",
  vars = val_vars,
  weight = "wgt"
)

# For precision evaluation
cdf_mn <- cdf_out$Mean

assign(paste0('estdat_2015_',i), data_2015)
assign(paste0('est_2015_',i), est_out)
assign(paste0('cdf_mn_2015_',i), cdf_mn)

}
```

```
##   stratum metric      value
## 1    None pielou 0.03648977
##   stratum metric      value
## 1    None pielou 0.04141772
```

### Change Analysis

Supporting Information for: Figure 8

Using the 3-year Florida standard based assessments for survey
periods 2015-2017 and 2017-2019, we estimated potential changes in
condition based on the differences observed in the percent of area
estimates generated for each para- meter.

Below is the raw code used to generate the change-analysis
results

```
# Changle Analysis

chng_dat <- bind_rows(estdat_3yr_2017, estdat_3yr_2019)

change_out <- change_analysis(
  chng_dat,
  siteID = "siteID",
  vars_cont = val_vars,
  vars_cat = fl_vars,
  surveyID = "surveyid",
  survey_names = c(2017, 2019),
  weight = "wgt"
)

# Extract change statistics
change_ests <- change_out$catsum
```

## Hexagon Sythesized Secondary Parameter Data

Supporting Information for: Figures 3 - 7

The following tables contain the hexagon synthesized data used to
inform the all pseudo-probability assessment demos. For each assessment,
a subset was extracted from this data set to meet the purpose of the
demonstration.

Click on a tab to switch between table views.

### FWIN 2015-2019 Data

### FWIN+NCCA 2015-2019 Data

## Assessment Estimate Tables

Supporting Information for: Figures 4 - 7

The following tables show probability condition estimates generated
by assessment analysis.

click on a tab to switch between table views.

### 2015 Condition Assessments

| Parameter | Category | Combined 2015 | FWIN 2015 |
| --- | --- | --- | --- |
| Chlorophyll a | Low | 5.6%+-%6.6 | NA |
| Chlorophyll a | Moderate | 30.6%+-%12.1 | 13.9%+-%6.3 |
| Chlorophyll a | High | 5.6%+-%6.6 | 2.8%+-%4.5 |
| Chlorophyll a | Not Sampled | 58.3%+-%14.4 | 83.3%+-%7.6 |
| Dissolved Oxygen | Low | 16.7%+-%10 | 16.7%+-%10.1 |
| Dissolved Oxygen | Moderate | 25%+-%11.6 | 13.9%+-%7.7 |
| Dissolved Oxygen | High | 8.3%+-%8.3 | NA |
| Dissolved Oxygen | Not Sampled | 50%+-%13.7 | 69.4%+-%8.8 |
| Enterococci | Low | 36.1%+-%13.1 | 8.3%+-%6.1 |
| Enterococci | Moderate | 2.8%+-%4.7 | 13.9%+-%10 |
| Enterococci | High | 2.8%+-%4.7 | NA |
| Enterococci | Not Sampled | 58.3%+-%14.4 | 77.8%+-%9.8 |
| PH | Moderate | 30.6%+-%11.6 | 22.2%+-%10 |
| PH | High | 19.4%+-%10.3 | 8.3%+-%5.1 |
| PH | Not Sampled | 50%+-%13.7 | 69.4%+-%8.8 |
| Salinity | Moderate | 13.9%+-%7.5 | 11.1%+-%6.3 |
| Salinity | High | 36.1%+-%13.8 | 19.4%+-%8.9 |
| Salinity | Not Sampled | 50%+-%13.7 | 69.4%+-%8.8 |
| Temperature | Moderate | 44.4%+-%13.4 | 30.6%+-%8.8 |
| Temperature | High | 5.6%+-%6.9 | NA |
| Temperature | Not Sampled | 50%+-%13.7 | 69.4%+-%8.8 |
| Total Nitrogen | Low | 8.3%+-%8.1 | 2.8%+-%4.6 |
| Total Nitrogen | Moderate | 33.3%+-%13.7 | 13.9%+-%7.7 |
| Total Nitrogen | High | 5.6%+-%6.3 | 5.6%+-%6.3 |
| Total Nitrogen | Not Sampled | 52.8%+-%14.1 | 77.8%+-%7.2 |
| Total Phosphorus | Low | 11.1%+-%8.8 | NA |
| Total Phosphorus | Moderate | 30.6%+-%10.4 | 22.2%+-%7.5 |
| Total Phosphorus | High | 8.3%+-%8 | 5.6%+-%6.3 |
| Total Phosphorus | Not Sampled | 50%+-%13.7 | 72.2%+-%7.3 |

### 2015-2019 Conditions Assessments

| Parameter | Category | FWIN 2015-2019 | Combined 2015-2019 |
| --- | --- | --- | --- |
| Chlorophyll a | Low | 4.4%+-%3.6 | 6.4%+-%4.1 |
| Chlorophyll a | Moderate | 44.4%+-%7.4 | 48.9%+-%7.2 |
| Chlorophyll a | High | 14.4%+-%5.8 | 14.9%+-%5.8 |
| Chlorophyll a | Not Sampled | 36.7%+-%6.9 | 29.8%+-%6.9 |
| Dissolved Oxygen | Low | 18.9%+-%6.5 | 18.1%+-%6.2 |
| Dissolved Oxygen | Moderate | 44.4%+-%8.8 | 46.8%+-%8.8 |
| Dissolved Oxygen | High | 15.6%+-%6 | 18.1%+-%6.3 |
| Dissolved Oxygen | Not Sampled | 21.1%+-%5.9 | 17%+-%6 |
| Enterococci | Low | 13.3%+-%5.5 | 23.4%+-%7.2 |
| Enterococci | Moderate | 22.2%+-%5.7 | 17%+-%5.5 |
| Enterococci | High | 8.9%+-%4.5 | 9.6%+-%4.7 |
| Enterococci | Not Sampled | 55.6%+-%6.3 | 50%+-%7 |
| PH | Low | 15.6%+-%5.6 | 14.9%+-%5.5 |
| PH | Moderate | 46.7%+-%8.9 | 47.9%+-%8.9 |
| PH | High | 12.2%+-%5.9 | 16%+-%6.5 |
| PH | Not Sampled | 25.6%+-%6.5 | 21.3%+-%6.5 |
| Salinity | Moderate | 56.7%+-%6.9 | 55.3%+-%6.6 |
| Salinity | High | 24.4%+-%6.6 | 29.8%+-%6.9 |
| Salinity | Not Sampled | 18.9%+-%5.7 | 14.9%+-%5.7 |
| Temperature | Low | 17.8%+-%7.1 | 17%+-%6.8 |
| Temperature | Moderate | 54.4%+-%8 | 57.4%+-%8.4 |
| Temperature | High | 6.7%+-%4.4 | 8.5%+-%4.5 |
| Temperature | Not Sampled | 21.1%+-%5.9 | 17%+-%6 |
| Total Nitrogen | Low | 13.3%+-%5.4 | 14.9%+-%5.7 |
| Total Nitrogen | Moderate | 38.9%+-%7.2 | 44.7%+-%7.5 |
| Total Nitrogen | High | 20%+-%5.7 | 19.1%+-%5.5 |
| Total Nitrogen | Not Sampled | 27.8%+-%6.7 | 21.3%+-%6.7 |
| Total Phosphorus | Low | 13.3%+-%4.6 | 17%+-%5.5 |
| Total Phosphorus | Moderate | 44.4%+-%6.8 | 45.7%+-%6.8 |
| Total Phosphorus | High | 16.7%+-%4.3 | 17%+-%4.4 |
| Total Phosphorus | Not Sampled | 25.6%+-%6.5 | 20.2%+-%6.6 |

### 3-Yr Survey Period Assessments

| Parameter | Category | Survey Period 2015-2017 | Survey Period 2017-2019 |
| --- | --- | --- | --- |
| Chlorophyll a | At/Below | 25%+-%7.4 | 42.9%+-%9.7 |
| Chlorophyll a | Above | 11.7%+-%5.3 | 19%+-%7.9 |
| Chlorophyll a | Not Sampled | 63.3%+-%5.3 | 38.1%+-%9.6 |
| Dissolved Oxygen | At/Below | 20%+-%7.6 | 9.5%+-%6 |
| Dissolved Oxygen | Above | 38.3%+-%9.6 | 61.9%+-%9 |
| Dissolved Oxygen | Not Sampled | 41.7%+-%7.5 | 28.6%+-%8.2 |
| Enterococci | At/Below | 30%+-%7.5 | 34.9%+-%8 |
| Enterococci | Above | 3.3%+-%4 | 4.8%+-%4.2 |
| Enterococci | Not Sampled | 66.7%+-%7.4 | 60.3%+-%8.5 |
| PH | At/Below | 3.3%+-%2.9 | 1.6%+-%2.6 |
| PH | Above | 53.3%+-%8.4 | 63.5%+-%8.7 |
| PH | Not Sampled | 43.3%+-%7.8 | 34.9%+-%8.3 |
| Total Nitrogen | At/Below | 38.3%+-%7.7 | 50.8%+-%9.3 |
| Total Nitrogen | Above | 8.3%+-%5.7 | 17.5%+-%6.4 |
| Total Nitrogen | Not Sampled | 53.3%+-%5.9 | 31.7%+-%9 |
| Total Phosphorus | At/Below | 48.3%+-%6 | 66.7%+-%9.6 |
| Total Phosphorus | Above | 3.3%+-%3.3 | 3.2%+-%3.7 |
| Total Phosphorus | Not Sampled | 48.3%+-%5 | 30.2%+-%8.9 |

### Change Assessment

| Survey.Period.1 | Survey.Period.2 | Parameter | Category | Difference | catsort |
| --- | --- | --- | --- | --- | --- |
| 2015-2017 | 2017-2019 | Chlorophyll a | At/Below | 17.9%+-%10.3 | 1 |
| 2015-2017 | 2017-2019 | Chlorophyll a | Above | 7.4%+-%8.3 | 2 |
| 2015-2017 | 2017-2019 | Chlorophyll a | Not Sampled | -25.2%+-%8.9 | 3 |
| 2015-2017 | 2017-2019 | Dissolved Oxygen | At/Below | -10.5%+-%8.1 | 1 |
| 2015-2017 | 2017-2019 | Dissolved Oxygen | Above | 23.6%+-%10.5 | 2 |
| 2015-2017 | 2017-2019 | Dissolved Oxygen | Not Sampled | -13.1%+-%8.4 | 3 |
| 2015-2017 | 2017-2019 | Enterococci | At/Below | 4.9%+-%9.4 | 1 |
| 2015-2017 | 2017-2019 | Enterococci | Above | 1.4%+-%2.5 | 2 |
| 2015-2017 | 2017-2019 | Enterococci | Not Sampled | -6.3%+-%9.2 | 3 |
| 2015-2017 | 2017-2019 | PH | At/Below | -1.7%+-%1.4 | 1 |
| 2015-2017 | 2017-2019 | PH | Above | 10.2%+-%8.7 | 2 |
| 2015-2017 | 2017-2019 | PH | Not Sampled | -8.4%+-%8.1 | 3 |
| 2015-2017 | 2017-2019 | Total Nitrogen | At/Below | 12.5%+-%10.1 | 1 |
| 2015-2017 | 2017-2019 | Total Nitrogen | Above | 9.1%+-%6 | 2 |
| 2015-2017 | 2017-2019 | Total Nitrogen | Not Sampled | -21.6%+-%8.1 | 3 |
| 2015-2017 | 2017-2019 | Total Phosphorus | At/Below | 18.3%+-%8.7 | 1 |
| 2015-2017 | 2017-2019 | Total Phosphorus | Above | -0.2%+-%3.3 | 2 |
| 2015-2017 | 2017-2019 | Total Phosphorus | Not Sampled | -18.2%+-%7.7 | 3 |

## Interactive Map

Click on/off map layers to see distribution of available secondary
data and hexagon overlays used to generate the pseudo-probabilisticaly
selected subset of data related to each of the assessment
demonstrations.

Map feature was produced using the R “leaflet” package.

Citation:

Cheng, J., Karambelkar, B., & Xie, Y. (2022). leaflet: Create
Interactive Web Maps with the JavaScript ‘Leaflet’ Library. https://CRAN.R-project.org/package=leaflet.

End
